# Senescent Endothelial Cells in Cerebral Microcirculation Are Key Drivers of Age‐Related Blood–Brain Barrier Disruption, Microvascular Rarefaction, and Neurovascular Coupling Impairment in Mice

**DOI:** 10.1111/acel.70048

**Published:** 2025-04-01

**Authors:** Boglarka Csik, Ádám Nyúl‐Tóth, Rafal Gulej, Roland Patai, Tamas Kiss, Jordan Delfavero, Raghavendra Y. Nagaraja, Priya Balasubramanian, Santny Shanmugarama, Anna Ungvari, Siva Sai Chandragiri, Kiana Vali Kordestan, Mark Nagykaldi, Peter Mukli, Andriy Yabluchanskiy, Sharon Negri, Stefano Tarantini, Shannon Conley, Tae Gyu Oh, Zoltan Ungvari, Anna Csiszar

**Affiliations:** ^1^ Vascular Cognitive Impairment, Neurodegeneration, and Healthy Brain Aging Program, Department of Neurosurgery University of Oklahoma Health Sciences Center Oklahoma City Oklahoma USA; ^2^ Oklahoma Center for Geroscience and Healthy Brain Aging University of Oklahoma Health Sciences Center Oklahoma City Oklahoma USA; ^3^ International Training Program in Geroscience, Doctoral School of Basic and Translational Medicine/Department of Public Health Semmelweis University Budapest Hungary; ^4^ First Department of Pediatrics Semmelweis University Budapest Hungary; ^5^ Eötvös Loránd Research Network and Semmelweis University (ELKH‐SE) Cerebrovascular and Neurocognitive Disorders Research Group Budapest Hungary; ^6^ International Training Program in Geroscience, Doctoral School of Basic and Translational Medicine/Department of Translational Medicine Semmelweis University Budapest Hungary; ^7^ Department of Public Health Semmelweis University Budapest Hungary; ^8^ Department of Health Promotion Sciences, College of Public Health University of Oklahoma Health Sciences Oklahoma City Oklahoma USA; ^9^ The Peggy and Charles Stephenson Cancer Center University of Oklahoma Health Sciences Center Oklahoma City Oklahoma USA; ^10^ Department of Cell Biology, College of Medicine University of Oklahoma Health Sciences Center Oklahoma City Oklahoma USA; ^11^ Department of Oncology Science, College of Medicine University of Oklahoma Health Sciences Center Oklahoma City Oklahoma USA

**Keywords:** aging, blood–brain barrier, cerebral microvascular endothelial cells, neurovascular coupling, senescence, vascular cognitive impairment and dementia

## Abstract

With advancing age, neurovascular dysfunction manifests as impaired neurovascular coupling (NVC), microvascular rarefaction, and blood–brain barrier (BBB) disruption, contributing to vascular cognitive impairment (VCI). Our previous research established a causal link between vascular senescence induced cerebromicrovascular dysfunction and cognitive decline in accelerated aging models. The present study examines whether chronological aging promotes endothelial senescence, adversely affecting neurovascular health, and whether senolytic therapies can enhance neurovascular function and cognitive performance in aged mice. We used transgenic p16‐3MR mice to identify and eliminate senescent cells and employed genetic (ganciclovir) and pharmacological (ABT263/Navitoclax) senolytic approaches. Evaluations included spatial memory performance, NVC responses, cortical microvascular density, BBB permeability, and detection of senescent endothelial cells via flow cytometry. Brain endothelial cells exhibited heightened sensitivity to aging‐induced senescence, undergoing senescence at a greater rate and earlier than other brain cell types, particularly during middle age. This microvascular endothelial cell senescence was associated with NVC dysfunction, microvascular rarefaction, BBB disruption, and deteriorating cognitive performance. On the other hand, senolytic treatments in aged mice improved NVC responses, BBB integrity, microvascular density, and learning capabilities. Notably, these findings suggest that the most effective time window for senolytic treatment is in middle‐aged mice, where early intervention could better prevent neurovascular dysfunction and mitigate age‐related cognitive impairment.

## Graphical abstract

Senolytic therapy restores neurovascular function, blood–brain barrier integrity, and cognitive performance in aged mice by targeting endothelial senescence. Findings highlight that brain microvascular endothelial cells are highly vulnerable to aging‐induced senescence, and early intervention in middle age may be the most effective strategy to prevent neurovascular dysfunction and cognitive decline.

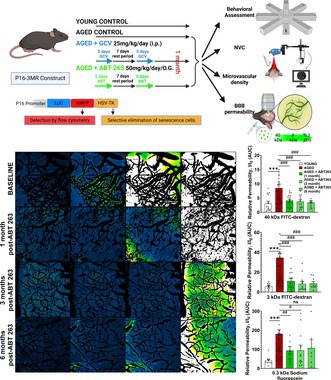

**Abbreviations**
3MR: trimodal fusion protein
ANGPT2: Angiopoetin‐2
AUC: area under the curve
BBB: blood–brain barrier
BCL: B‐cell lymphoma
BSA: bovine serum albumin
BubR1: budding uninhibited by benzimidazole‐related 1
CBF: cerebral blood flow
CDKN: cyclin‐dependent kinase inhibitor
CMVEC: cerebromicrovascular endothelial cell
CXCL: C‐X‐C motif chemokine ligand
DLL4: delta‐like ligand 4
EndoMT: endothelial to mesenchymal transition
FACS: fluorescent activated cell sorting
fEPSP: field excitatory postsynaptic potential
FITC: fluorescein isothiocyanate
GCV: ganciclovir
GEM: gel bead‐in‐emulsion
GLAST: glutamate aspartate transporter
GOPB: gene ontology biological processes
IL: interleukin
LTP: long‐term potentiation
MEA: multi‐electrode array
MMP: matrix metalloproteinase
NIH: National Institute of Health
NO: nitric oxide
NOTCH: neurogenic locus notch homolog protein
NVC: neurovascular coupling
OUHSC: University of Oklahoma Health Sciences Center
PBS: phosphate buffered saline
PCA: principal component analysis
PDGF: platelet‐derived growth factor
PTPRM: protein tyrosine phosphatase receptor type M
RAWM: radial arms water maze
RFP: red fluorescent protein
SASP: senescence‐associated secretory phenotype
scRNA‐seq: single‐cell RNA sequencing
SEM: standard error of mean
SMAD: suppressor of moters against decapentaplegic
TGF‐β: transforming growth factor β
THBS: thrombospondin
Tie2: tyrosine kinase with immunoglobulin and epidermal growth factor homology domains 2
TNF‐α: tumor necrosis factor α
VCI: vascular cognitive impairment
VEGF: vascular endothelial growth factor
VOI: volume of interest
WGA: wheat germ agglutin
Wnt: wingless‐related integration site

## 1 Introduction

The burgeoning prevalence of age‐related vascular cognitive impairment (VCI), marked by cognitive deficits stemming from various cerebrovascular pathologies, is emerging as a pressing public health issue (Johnson 2023; Iadecola et al. 2019; Toth et al. 2017; Gorelick et al. 2016; Mahinrad et al. 2023). With demographic shifts towards an aging population, the percentage of individuals aged 65 and above has exceeded 20% in countries such as Japan and many within the European Union, with projections indicating a further increase in the coming decades (Eurostat 2022; World Health Organization 2023). This demographic evolution is pivotal, as a significant portion of this aging population is expected to experience VCI, thereby exacerbating functional dependence issues and imposing considerable socioeconomic challenges (GBD 2019 Dementia Forecasting Collaborators 2022).

At the heart of the pathogenesis of VCI lie intricate aging‐related microvascular alterations within the brain, notably neurovascular dysfunction (Toth et al. 2017; Sweeney et al. 2018a, 2018b). This results in a compromised neurovascular coupling (NVC, or functional hyperemia), a crucial mechanism for adjusting regional blood flow to align with the metabolic needs of active brain areas (Toth et al. 2017). Moreover, microvascular rarefaction, the progressive loss of capillaries, exacerbates these neurovascular alterations, contributing significantly to the reduction in cerebral blood flow and the compromised delivery of essential nutrients and oxygen (Toth et al. 2017; Nyul‐Toth et al. 2021). Concurrently, age‐related disruption of the blood–brain barrier (BBB), a selective permeability barrier essential for maintaining brain homeostasis and protecting neural tissue from toxic substances and pro‐inflammatory factors, further exacerbates cognitive decline (Sweeney et al. 2018a, 2018b). Age‐related BBB disruption has been causally linked to neuroinflammation, synaptic dysfunction, and white matter injury (Sweeney et al. 2018a, 2018b). Together, the age‐related impairment of NVC and BBB integrity is critical in undermining brain health and function, setting the stage for the development of VCI (Toth et al. 2017; Sweeney et al. 2018a, 2018b).

The etiology of age‐associated cerebromicrovascular pathologies is multifaceted, with oxidative stress‐mediated DNA damage leading to cellular senescence emerging as a pivotal mechanism (Fulop et al. 2018; Kiss et al. 2020a; Tarantini et al. 2021; Faakye et al. 2023). Specifically, cerebromicrovascular endothelial cells are highly susceptible to this form of damage, predisposing them to senescence (Ungvari et al. 2013). Senescent cerebromicrovascular endothelial cells exhibit hallmark features of cellular senescence, including cell cycle arrest, significant morphological alterations, and a senescence‐associated secretory phenotype (SASP) (Ungvari et al. 2013; Kiss et al. 2020b). The SASP is characterized by the secretion of pro‐inflammatory cytokines and matrix metalloproteinases (MMPs), which contribute to the microvascular and cognitive impairments observed both in aging and conditions marked by accelerated microvascular aging (Faakye et al. 2023; Ungvari et al. 2013; Toth et al. 2015a). Preclinical studies have increasingly affirmed the role of senescent endothelial cells and their SASPs in cerebromicrovascular pathologies (Tarantini et al. 2021; Faakye et al. 2023; Ahire et al. 2023; Gulej et al. 2023a; Yabluchanskiy et al. 2020). Research utilizing genetically modified mice to deplete senescent cells expressing the senescence marker p16^*INK4A*^ has shown notable improvements in lifespan, healthspan, microvascular function, and cognition (Faakye et al. 2023; Baker et al. 2016, 2011; Jeon et al. 2017; Abdul‐Aziz et al. 2019; Kim et al. 2019; Patil et al. 2019; Farr et al. 2017; Xu et al. 2015; Roos et al. 2016; Baar et al. 2017). This underscores the detrimental effects of cellular senescence on both cerebral and microvascular aging. Our recent work has further evidenced that senolytic treatments not only enhance endothelial regulation of cerebral blood flow (CBF) but also alleviate hypertension‐induced cerebral microhemorrhages in aged mice (Tarantini et al. 2021; Faakye et al. 2023). Additionally, our experiments employing gamma irradiation to induce endothelial senescence in mice have extended these observations, demonstrating that the removal of senescent cells can reverse neurovascular dysfunction, thereby rescuing NVC responses and restoring BBB integrity (Yabluchanskiy et al. 2020). Despite these advancements, the specific impact of endothelial senescence on NVC responses and BBB function in naturally aging mice remains to be fully elucidated.

This study is designed to investigate the hypothesis that cerebromicrovascular endothelial senescence contributes to impaired NVC, microvascular rarefaction, and compromised BBB integrity in the aging brain. We hypothesize that the elimination of senescent cells will enhance NVC responses, increase capillarization, restore BBB functionality, and consequently improve cognitive performance in aged mice. To test these hypotheses, we employed both young and aged transgenic p16‐3MR mice, which allow for the identification and targeted removal of senescent cells (Ahire et al. 2023; Gulej et al. 2023a; Yabluchanskiy et al. 2020). To assess the direct impact of age‐related senescence on neurovascular dysfunction, capillarization, and BBB integrity, we depleted senescent cells in aged animals using either the p16‐3MR transgene activation or the administration of Navitoclax/ABT263, a senolytic agent known to target B‐cell lymphoma (BCL) 2/BCL‐xL (Chang et al. 2016). We conducted comprehensive evaluations of NVC responses using laser speckle contrast imaging and assessed capillary density and BBB permeability to fluorescent tracers via two‐photon microscopy through a chronic cranial window. Additionally, the presence of senescent microvascular endothelial cells was verified using flow cytometry and single‐cell RNA sequencing (scRNA‐seq), providing a multifaceted approach to understanding the mechanisms underlying neurovascular aging and identifying potential therapeutic targets for mitigating aging‐associated cognitive decline.

## 2 Materials and Methods

### 2.1 Experimental Animal Models and Housing Conditions

**FIGURE 1:**
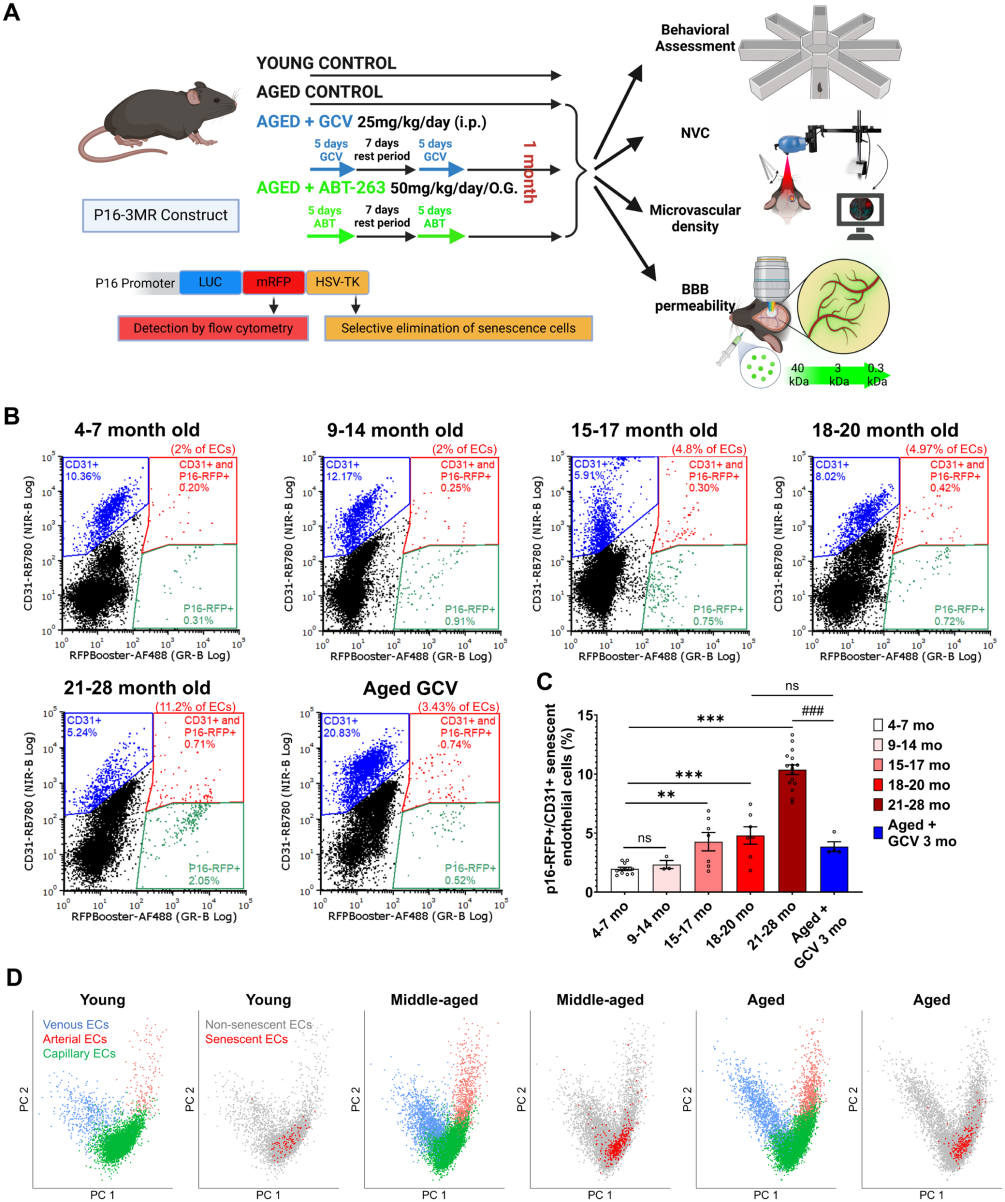
Impact of aging and senolytic treatments on cerebromicrovascular endothelial senescence in the mouse brain. (A) Schematic of the experimental timeline illustrating the assessment of senolytic treatments in aged p16‐3MR mice. The study delineates four groups for comparison: Young Control, Aged Control, Aged +senolytic treatment with GCV, and Aged +senolytic treatment with ABT263. Assessments conducted include learning performance, neurovascular coupling (NVC) responses, blood–brain barrier (BBB) permeability, and cerebromicrovascular density, the latter two being examined through intravital two‐photon microscopy (TPM), providing a comprehensive analysis of the senolytic treatments' impact on cerebromicrovascular health in the context of aging. (B) Flow cytometric analysis highlights RFP+/CD31+ senescent and RFP‐/CD31+ non‐senescent endothelial cells in brain‐derived single‐cell suspensions from p16‐3MR mice of different ages. Representative dot plots illustrate RFP‐Booster AlexaFluor488 fluorescence (indicative of p16‐3MR expression) against CD31 staining (RB780), showing the populations of endothelial cells (RFP‐, CD31+), endothelial senescent cells (RFP+, CD31+, and non‐endothelial senescent cells (RFP+, CD31‐). (C) presents summarized data on the prevalence of senescence within endothelial cells across different age groups, with values expressed as mean ± SEM (*n* ≥ 4 for each group). A significant age‐related increase in endothelial cell senescence burden within the mouse brain is evident when compared to younger counterparts (**p < 0.01, ***p < 0.001 vs. Young; ##*p* < 0.001 vs. Aged (21–28‐month‐old)). (D) Principal component analysis (PCA) of scRNA‐seq data from brain endothelial cells of young (6‐month‐old), middle aged (16‐month‐old) and aged (24‐months‐old) mice. The PCA identifies subclusters of endothelial cells—capillary, venous, and arterial—based on distinct gene expression patterns. The visualization showcases these endothelial cell subtypes, color‐coded by phenotype. Senescent endothelial cells are highlighted (red). Senescent endothelial cells, identified based on high expression of senescence markers, are prominently marked in red. This panel underscores the presence and distribution of senescent cells across different endothelial cell types, illustrating the pervasive impact of aging on microvascular endothelial cell senescence.

Our study employed p16‐3MR transgenic male and female mice to investigate the role of senescent cells in age‐related NVC impairment, microvascular rarefaction, and BBB dysfunction. p16‐3MR mice (Yabluchanskiy et al. 2020; Demaria et al. 2014) carry a trimodal fusion protein (3MR) under the control of the p16^*Ink4a*^ promoter. 3MR contains functional fragments of Renilla luciferase, which allows us to detect senescent cells in living animals, monomeric red fluorescent protein (RFP), which enables us to identify senescent cells from tissues via flow cytometry, and the herpes simplex virus thymidine kinase, which allows us to selectively kill p16‐positive senescent cells by administering the prodrug ganciclovir (Figure 1A). Previous studies have extensively characterized this model (Ahire et al. 2023; Demaria et al. 2017). To characterize the time course of age‐related changes in NVC, BBB, and cortical capillary density and the progression of senescent cell accumulation, mice of different ages were used.

Animals were housed under a consistent light–dark cycle, with 12 h of light followed by a 12 h dark period and provided *ad libitum* access to water and a standard AIN‐93G diet. Initially, the mice were housed under specific pathogen‐free conditions within the Rodent Barrier Facility at the University of Oklahoma Health Sciences Center (OUHSC). Subsequently, for the duration of the experimental procedures, they were relocated to the standard rodent colony at OUHSC. Our research strictly adhered to the ethical guidelines outlined in the National Institutes of Health (NIH) Guide for the Care and Use of Laboratory Animals (NIH Publications No. 8023, revised 1978). Consistent with these guidelines, we implemented rigorous measures—both institutional and tailored to our study—to minimize potential distress or discomfort to the animals involved. Our commitment to ethical research practices involved ongoing assessments and adjustments to enhance animal welfare, demonstrating our dedication to responsible and compassionate scientific inquiry. All animal protocols were approved by the Institutional Animal Care and Use Committee of the OUHSC.

### 2.2 Senolytic Treatments

In our investigation to elucidate the pathophysiological impact of senescent cells on aging, we categorized aged mice into three distinct groups through random assignment. Two of these groups were subjected to senolytic treatments at 18 months of age: one received ABT263 (Navitoclax, Chemgood, C‐1009, administered at a dosage of 50 mg/kg daily via oral gavage) and the other was treated with ganciclovir (GCV [TSZCHEM, RG001, > 99%]; intraperitoneally, at a dosage of 25 mg/kg daily in PBS). These treatments were applied for a duration of 5 days, across two treatment cycles, with a 2‐week resting period between cycles. The remaining group functioned as the vehicle control, receiving no active senolytic agents. The selection of dosages, along with the routes and methods of administration, was informed by thorough evaluations in prior studies that aimed to determine the most effective approach for achieving senescent cell clearance. The efficacy of these senolytic treatments has been confirmed through flow cytometry analysis, which verified the successful reduction of senescent cell populations following treatment (Ahire et al. 2023; Gulej et al. 2023a; Yabluchanskiy et al. 2020).

### 2.3 Cognitive Function Testing With Radial Arms Water Maze (RAWM)

To determine how depletion of senescent cells affects cognitive function, spatial memory and long‐term memory were tested by assessing performance in the radial arms water maze at 1 month after senolytic treatment, following our published protocols (Ahire et al. 2023). The maze comprises eight arms, each leading to a submerged escape platform located at the end of one arm. Opaque water, achieved by adding food coloring, filled the maze. Surrounding the maze were privacy blinds, supplemented with extra‐maze visual cues, while intra‐maze visual cues were positioned at the arm ends. Video tracking directly above the maze, coupled with Ethovision software (Noldus Information Technology Inc., Leesburg, VA, USA), was employed for monitoring mice, with experimenters unaware of the experimental conditions. During each daily session, mice underwent two blocks, each encompassing four consecutive acquisition trials, to learn the submerged escape platform's location. Starting in an arm without the platform, mice were given up to 1 min to locate the escape platform, spending 30 s on it after each trial. The platform remained in the same arm for every trial. Over 3 days of training, mice exhibited gradual performance improvement. Errors were charged when a mouse entered an incorrect arm, defined as having all four paws within the distal half of the arm. After the group acquired the task, mice were placed in their home cage for 7 days, followed by a recall/probe trial on day 10. On day 11 (reversal/extinction), mice were tested for their ability to relearn the task, with the platform relocated to a different arm not adjacent to or diametrically positioned to the previous location. The mice underwent testing for two session blocks, with the second block, comprising four trials, used for comparison.

### 2.4 Assessment of NVC Responses Using Laser‐Speckle Flowmetry

NVC responses were evaluated following established protocols (Ahire et al. 2023). For each experimental group, mice were anesthetized using isoflurane, initiated at a 3% concentration for induction and subsequently maintained at 1%. Endotracheal intubation was performed with a 20Gx1 catheter (Ref: 266741, Exel, CA, USA), followed by mechanical ventilation with a MousVent G500 system (Kent Scientific Co, Torrington, CT). A thermostatically controlled heating pad (Kent Scientific Co, Torrington, CT) was used to maintain the core temperature of the mice at a steady 37°C. End‐tidal CO_2_ was controlled between 3.2% and 3.7% to keep blood gas values within the physiological range, as described (Tarantini et al. 2015; Toth et al. 2015b). Mice were immobilized and placed on a stereotaxic frame (Leica Microsystems, Buffalo Grove, IL); the scalp and periosteum were pulled aside, and the skull was gently thinned using a dental drill while cooled with dripping buffer. A fine layer of nail polish was applied to the skull to enhance the optical interface. A laser speckle contrast imager (Perimed, Järfälla, Sweden) was then positioned approximately 10 cm above the skull. NVC responses were induced by sequential bilateral whisker stimulation at a frequency of 5 Hz for 30 s. Differential perfusion maps of the brain surface were captured. This measurement cycle was conducted in eight rounds, separated by 5 min intervals. The resulting changes in CBF were averaged and reported as a percentage increase from baseline values.

### 2.5 Quantification of Senescent Endothelial Cell Burden via Flow Cytometry

We used cells obtained from the single‐cell suspensions from the brain samples derived from young, middle aged, and aged p16‐3MR mice to analyze senescent cell burden, following our published protocol with slight modifications (Ahire et al. 2023). Animals were euthanized and transcardially perfused with ice‐cold phosphate‐buffered saline (PBS) for 12 min. The brains were collected and transferred to ice‐cold PBS in a sterile Petri dish. Subsequently, the olfactory bulb, cerebellum, and brainstem were excised using a sterile scalpel, and meningeal vessels were removed by rolling the brain on Whatman paper. The brain tissue was finely chopped, placed in a 50 mL conical tube, and centrifuged (50 × g, 5 min, 4°C). The samples were washed with sterile PBS, and then digestive enzymes (50 μL collagenase, 2 μL elastase [Sigma, E0127], 2 μL dispase I [Sigma, D4818], and 3 μL hyaluronidase [Sigma, H4272]) were added, followed by incubation in a rotating incubator for 45 min at 37°C. The tissue was carefully dissociated by resuspending it first with a 10 mL and then a 5 mL pipette tip. Following this initial dissociation, the tissue suspension was sequentially filtered through 100 μm and then 30 μm nylon mesh filters (Miltenyi Biotec) to achieve a homogeneous single‐cell suspension. The cells were then centrifuged (300 × g, 10 min, 4°C) and resuspended in 3.1 mL of PBS. Afterward, 0.9 mL of Debris Removal Solution (Miltenyi Biotec) was added, and tubes were mixed by inverting five times. Afterward, 4 mL of PBS was slowly overlaid, and the samples were centrifuged at 3000 × g for 10 min with full brake and full acceleration based on the manufacturer's recommendation. This resulted in the formation of a top clear buffer layer, a white interphase containing myelin debris, and a bottom clear layer containing pelleted cells. The top and middle layers were removed, and the cells were washed in sterile PBS. The samples were centrifuged (1000 × g, 10 min, 4°C), the supernatant was discarded, the cells were resuspended in 1 mL of MACS Buffers (Miltenyi Biotec) and their concentration and quality were assessed using a disposable hemocytometer (C‐Chip, DHC‐N01, InCyto). For each staining, 50,000 cells were transferred to the specific wells on a 96‐well plate, and filtered MACS buffer was added to the volume of 130 μL. The samples were then blocked with 10 μL of 7.5% bovine serum albumin (BSA) in PBS (~0.5% final concentration, 15 min, at room temperature). Subsequently, surface staining was performed. Anti‐CD31 antibody (1:150, RB780, cat#:569358, BD Biosciences) was used to stain endothelial cells. Samples were stained for 60 min at room temperature, on a shaker, protected from light. Samples were washed three times using MACS buffer, and then fixed using 2% paraformaldehyde (15 min, at room temperature, protected from light). Fixed cells were washed with MACS twice and resuspended in 0.5% Tween 20 in PBS to permeabilize cells for intracellular staining (30 min, at room temperature, protected from light). Permeabilized cells were stained with RFP‐Booster (1:150, AlexaFluor‐488, Chromotek; US‐QUO201590, 0.5 g/L) for 45 min, at room temperature, protected from light. The RFP‐Booster allows for the detection of senescent cells that express the RFP‐containing 3MR construct under the control of the p16^*Ink4a*^ promoter (p16‐RFP). Samples were washed twice with 0.5% Tween 20 in PBS and resuspended in 200 μL of MACS buffer for acquisition. The prevalence of p16‐RFP^+^ cells in single‐cell brain lysates was determined using a Guava EasyCyte BGR HT Flow Cytometer (Luminex). Antibody titration experiments were conducted to determine optimal concentrations of RFP Booster, and anti‐CD31, anti‐CD11b (1:200, PE‐Cy7, #552850, BD Biosciences, USA), anti‐CD140b (1:75, APC, Miltenyi Biotech, USA), and anti‐glutamate aspartate transporter (GLAST, 1:150, APC, Miltenyi Biotech, USA) antibodies. Non‐stained samples and fluorescence minus one (FMO) controls were analyzed to determine optimal compensation settings and gates for data analysis. To assess the ratio of senescence within endothelial cells, the number of double‐positive cells (p16‐RFP^+^/CD31^+^) was divided by the number of all CD31^+^ endothelial cells. The senescence burden within other cell types (microglia, pericytes, and astrocytes) was assessed in the same way. Flow cytometry data analysis and the generation of representative plots were performed using FCS Express 7 (De Novo Software) analysis software.

### 2.6 Identification of Senescent Microvascular Endothelial Cells Using scRNA‐seq

To discern senescent cells within the brains of both young and aged mice, we employed a single‐cell transcriptomics approach, as previously describe (Kiss et al. 2020b; Ahire et al. 2023). Our methodology involved the utilization of a gel bead‐in‐emulsion (GEM) droplet sequencing technique, which is particularly suited for the comprehensive and unbiased examination of a vast number of brain cells. This technique allowed us to classify cerebromicrovascular endothelial cells, among other brain cell types, by their distinct gene expression profiles. By comparing these profiles to known transcriptomic signatures of cellular senescence, we were able to accurately identify senescent microvascular endothelial cells present, employing criteria and methods established in prior research (Kiss et al. 2020b; Ahire et al. 2023; Gulej et al. 2023b).

After perfusion with ice‐cold PBS, brains from both young and aged mice (*n* = 5 per group) were promptly extracted, washed in ice‐cold PBS, and dissected into approximately 1 mm (Toth et al. 2017) pieces. We prepared single‐cell suspensions from these brain tissues, adapting the protocol utilized in our flow cytometry analysis, with specific modifications for this application. Initially, the brain samples underwent digestion and purification using Debris Removal Solution (Miltenyi Biotec). Subsequently, the cell pellets were resuspended in PBS containing 0.1% BSA and stained with SYTOX Green Nucleic Acid Stain (Invitrogen) for the identification of live cells. We employed the WOLF Cell Sorter (NanoCellect) for fluorescent activated cell sorting (FACS) at low pressure, efficiently removing cellular debris and dead cells to enrich the single‐cell suspension with viable cells. This preparation was maintained on ice pending sequencing, a crucial step to ensure the integrity and high quality of cells suitable for transcriptomic analyses. All samples were isolated and processed concurrently to construct stable cDNA libraries. After sorting, cells were centrifuged (2200 rpm for 10 min at 4°C) and resuspended in 30–50 μL of 0.04% BSA, adjusted according to the quantity of sorted cells. These cells were then introduced into a Chromium Single Cell 3′ Chip (10× Genomics, Pleasanton, California) and processed as per the guidelines provided by the manufacturer. The construction of libraries utilized the Chromium Single Cell 3′ Library & Gel Bead Kit v2 (Catalog# 120267, 10× Genomics, Pleasanton, California). Subsequent to pooling the libraries based on their molar concentrations, the combined library was sequenced using a high‐output lane on the NovaSeq 6000 system (Illumina, San Diego, California). Sample de‐multiplexing, barcode processing, read alignment and filtering, and the generation of feature‐barcode matrices were conducted using the Cell Ranger (v3.0.2) software suite from 10× Genomics, following the manufacturer's protocol. Mapping of reads was carried out against the mm10 mouse transcriptome reference (version 1.2.0) provided by 10× Genomics.

Downstream analyses of the Cell Ranger outputs were conducted using the Seurat (version 4.1) toolkit, which is integrated within an R package environment (R version 4.1.1). The first phase of data refinement involved eliminating cells of low quality, specifically those with either exceedingly high or low counts of unique genes, as well as those exhibiting a high proportion of reads mapping to the mitochondrial genome (exceeding 15% of all reads), as these indicators could skew the analysis. To mitigate technical variability, data normalization was carried out using the SCTransform algorithm, further enhanced by the glmGamPoi method. This step involved adjusting for the variable “percentage of reads mapping to the mitochondrial genome”, with other parameters set to their default values. Utilizing the top 3000 variable features identified by SCTransform, principal component analysis (PCA) was executed to distill the data, employing the default settings of the Run‐PCA function. Cell clustering proceeded with the FindNeighbors and FindClusters functions, leveraging the top 25 PCA components. This unbiased approach utilized the Louvain clustering algorithm with a resolution parameter set at 0.065, facilitating the categorization of cells into distinct clusters based on their gene expression profiles. Clusters constituting less than 3% of the total cell population were excluded from subsequent analyses to focus on the most representative cell types. The identity of cell clusters was determined by comparing their gene expression against known markers for canonical cell types, with a significant presence of endothelial cells detected.

Our objective was to pinpoint senescent cells through their distinct gene expression signatures. To this end, we characterized senescence‐associated gene expression at the single‐cell level by applying a modified enrichment score for each cell (Subramanian et al. 2005), utilizing the AUCell algorithm. This approach leverages a curated set of core senescence‐associated genes, assembled from relevant literature, as we have previously detailed (Ahire et al. 2023). Specifically, within each cell, genes are ranked according to their expression levels, and an “Area Under the Curve” (AUC) analysis is conducted. This analysis assesses the enrichment of a crucial subset of the input gene set among the genes expressed by each cell (Ahire et al. 2023). Following established protocols, we set a threshold based on the distribution of cell‐specific senescence enrichment scores, enabling the classification of each cell as either senescent or non‐senescent.

Subsequent analyses focused on endothelial cells, analyzed using PCA, and further divided into sub‐clusters, all within the Seurat (version 4.1) framework (Ahire et al. 2023). The RunPCA function, with default settings, facilitated the PCA embedding. For finer sub‐clustering, we selected the top 20 PCA dimensions and adjusted the resolution parameter to 0.2 for both the FindNeighbors and FindClusters functions. This process identified five distinct sub‐clusters of endothelial cells. These were then grouped into three categories based on canonical marker genes: endothelial cells from arteries (two clusters), veins (two clusters), and capillaries (one cluster), providing a nuanced view of endothelial cell heterogeneity in the context of senescence (Ahire et al. 2023). The previously published list of senescence‐related gene set (Kiss et al. 2020a) and the SenMayo gene set have been used to determine endothelial senescence (Saul et al. 2022).

### 2.7 Single Cell Transcriptome Analysis

Initially, raw fastq files were quality checked. Then, cellranger was utilized to generate aligned and demultiplex count files. The cellranger outcome, filtered_feature_bc_matrix, was loaded into the Seurat environment using the “Read10X” function. For normalization, the scale factor was set at 10,000, and the “LogNormalize” method was used. After finding variable features, “ScaleData” and “RunPCA” commands were used. Clustering and dimensionality reduction were performed using “FindNeighbors” and “FindClusters” to identify cell clusters, followed by “RunUMAP” for visualization. For doublet detection, DoubletFinder was applied. The expected doublet proportion was set at 8.7% of the total cells, and potential doublets were excluded for post‐analysis. Cell type definition was completed using the scMRMA package and manual gene check‐up. For further clustering, endothelial cells, the endothelial cells were bioinformatically extracted. Then, the previous marker genes (Walchli et al. 2024) were used. Gene signatures were extracted from annotation markers and grouped by cell type. The top 20 genes per cell type were selected based on logFoldChange, forming a list of signature genes cell rankings were computed. The “AUCell_calcAUC” function was then applied to calculate the AUC scores for each cell based on the defined signature list. The resulting AUC matrix was transposed and used for cell annotation. Cells were assigned to the signature with the highest AUC score if the maximum score exceeded 0.25, and unassigned cells were further examined manually with known marker genes. To visualize the data, the ShinyCell was utilized. To examine the cell‐to‐cell communication, The CellChat was employed to visualize the cross‐talk among cell types.

### 2.8 Cranial Window Surgery Protocol

Our laboratory employs an approved and standardized procedure for cranial window surgery, as extensively detailed in our prior publication (Nyul‐Toth et al. 2021). In brief, mice were initially anesthetized with isoflurane (3%–4% for induction, 2%–3% for maintenance, at a flow rate of 0.6–0.8 L/min) and positioned on a stereotactic stage under a Zeiss Stemi 2000 stereomicroscope. Ophthalmic eye ointment was applied to protect their eyes, and for sterility, head hair was removed, and the skin was thoroughly cleaned with povidone‐iodine surgical scrub and a 70% ethanol wipe. Once the mouse reached a fully anesthetized state, indicated by the absence of paw or tail reflexes, an oval section of skin covering the skull was carefully excised using pointed‐end scissors. The skull surface was gently scraped with a disposable scalpel. Lidocaine (2% saline solution) was then applied to the skull, allowed to sit for a couple of minutes, and followed by drilling. The craniotomy was performed over the somatosensory cortex, 2–3 mm posterior to the coronal suture and lateral to the sagittal suture, with a diameter of approximately 4 mm. Drilling involved creating a shallow circular outline, gradually thinned with the drill. Once the skull within the groove was thin enough, the circular skull piece was detached using fine forceps under a drop of sterile saline. Gelatin sponges soaked in sterile saline were employed for hemostasis if bleeding occurred (Dengofoam, ref.: 600034, Dengen Dental, WY, USA). A glass round coverslip (⌀5 mm, ref.: 64–0700, Warner Instruments, MA, USA) was disinfected, wiped clean, and rinsed with sterile saline; then with a drop of sterile saline underneath, it was carefully placed over the craniotomy. A small amount of cyanoacrylate glue was applied around the window to attach it to the bone. To secure the edges of the glass window, a layer of acrylic cement (Dental Cement, ref.: 51459, Stoelting, IL, USA) was added around the coverslip and on the exposed skull. Following the hardening of the acrylic layer, the mouse received buprenorphine (1 mg/kg body weight, sc injection, Buprenorphine ER, 1 mg/mL, ZooPharm, WY, USA) for pain management and enrofloxacin (10 mg/kg body weight, sc injection, 2.27%, Baytril, Elanco, IN, USA) to prevent infection. Subsequently, the mouse was transferred to a clean cage and closely observed until fully awake. Hydrogel (70–01–5022, ClearH2O, ME, USA) and food pellets were provided on the cage floor to facilitate recovery. The mouse's well‐being and recovery were monitored closely for up to a week post‐surgery, and necessary actions were taken in case of rare complications, with consultation from the OUHSC veterinarians.

### 2.9 Measurement of Blood–Brain Barrier Integrity and Microvascular Density Using Intravital Two‐Photon Microscopy

In each group, mice equipped with chronic cranial windows underwent isoflurane anesthesia (3% induction, 2% maintenance, with a flow rate of 0.6–0.8 L/min) and the head was securely positioned with ear bars into a stereotaxic frame. After adding eye ointment, the setup was moved under a Fluoview FV1000 two‐photon microscope, equipped with a water immersion objective (XLPLN25XWMP, 25×, 1.05 NA; Olympus, Tokyo, Japan) and an 800‐nm laser line for excitation. Emitted light was detected by PMT detectors using three filter sets (420–460, 495–540, and 575–630 nm; (Nyul‐Toth et al. 2021)). Subsequently, Alexa Fluor594‐conjugated Wheat Germ Agglutin (WGA‐AF594, 1 mg/mL, 4 μL/g body weight, ref.: W11262, ThermoFisher Scientific, MA, USA) was retro‐orbitally injected for blood vessel visualization, and Z‐stacks with 5 μm z‐intervals were captured to establish a baseline. FITC‐conjugated dextrans of decreasing molecular weights (70‐, 40‐, 10‐, and 3 kDa FITC‐dextrans, 4 μL per gram of body weight, 2 mg/mL; ref.: D1823, D1845, D1821, D3305, respectively, ThermoFisher Scientific, MA, USA) were then retro‐orbitally injected. Each tracer was injected alone sequentially after each other, and multiple 15‐min time Z‐stacks were acquired (one Z‐stack per minute) after each injection, resulting in a hyperstack. After the 15 min acquisition period had elapsed, the next smaller tracer was injected, and the 15 min image acquisition period was repeated.

### 2.10 Analysis of Blood‐Brain Barrier Measurements and Microvascular Density Based on Multi‐Photon Imaging

For the analysis of multi‐photon z‐stack images, we employed the ImageJ software (version 1.53 t, NIH, USA) based on an enhanced version of our previously established image processing and analysis protocol (Nyul‐Toth et al. 2021). The procedure commenced with the concatenation of the baseline image and the subsequent time‐stacks, each comprising 15 post‐tracer images, resulting a time‐Z‐stack of the whole measurement. To enhance the accuracy of our analysis, three‐dimensional alignment was performed on these images using the “Correct 3D drift” plugin available in ImageJ. To focus of our analysis on the relevant depth range of 50–150 μm, the uppermost layer of meningeal blood vessels within the first 50 μm from the surface was excluded from the z‐stacks. The images were then transformed into two‐dimensional representations utilizing the “Max Intensity” projection. Binary vascular masks were created using the *Trainable Weka Segmentation* plugin classified vasculature and extravascular spaces on the maximal intensity projection images. The binary masks for microvascular complexity analysis excluded autofluorescent particles (“*Analyze particles*”, size ≤ 20 px^2^, circularity 0–1), to prevent misidentification as vasculature. The edges of the vasculature in these masks were smoothed using “*Maximum*” and “*Minimum*” filter functions in ImageJ. The processed binary images were measured for vascular area coverage as vascular density (%) and then skeletonized for the analysis of total vessel length as a measure of vascular length density (μm/μm^2^) in the volume of interest (VOI).

To quantify BBB permeability, we used a “relative permeability changes over‐time” paradigm. For this we used the concatenated image time‐Z‐stacks and subtracted the maximal intensity projection images to obtain the extravascular fluorescent intensity changes over baseline, measured in the green channel (FITC‐dextrans). The masks of the vasculature created using the “*Trainable Weka Segmentation*” plugin, included the autofluorescent signal, which was subsequently subtracted from the brain parenchyma to refine the analysis. The cumulative changes in green fluorescence (*I* [a.u.]) compared to the baseline (*I*_*0*_ [a.u.]) for each tracer were measured for each experimental animal and plotted against time, giving a function, and the AUC has been used as the relative permeability (*I/I*_*0*_) of the blood–brain barrier in the VOI.

### 2.11 Electrophysiology

Slice preparation: Both male and female mice at 4–32 months of age were used in this study. To collect slices, mice were decapitated, and their brains were quickly removed and cooled with oxygenated ice‐cold sucrose slicing solution containing (in mM) 240 sucrose, 25 NaCl, 2.5 KCl, 1.25 NaH_2_PO_4_, 26 NaHCO_3_, 0.4 Ascorbic acid, 10 D‐glucose, 10 MgCl_2_, and 2 Sodium Pyruvate (oxygenated with 95% O_2_/5% CO_2_). Appropriate portions of the brain were trimmed, and the tissue block containing the hippocampus was glued onto the stage of a vibrating microtome (HM650, Thermo Fisher Scientific) with the support of an agar block. Horizontal brain slices (350 μm thick) were collected from the vibrating microtome in oxygenated cold sucrose slicing solution. The slices were transferred to a holding chamber containing oxygenated artificial cerebrospinal fluid (ACSF) containing the following (in mM): 126 NaCl, 2.5 KCl, 1.25 NaH_2_PO_4_, 26 NaHCO_3_, 1.0 CaCl_2_, 1.0 MgCl_2_, 2 Sodium Pyruvate, 0.4 Ascorbic acid, and 10 D‐glucose (final pH 7.4). The slices were allowed to recover for at least 1 h in ACSF at room temperature.

Multi‐Electrode Array (MEA) Recordings: For recording extracellular field potentials, the slice was transferred to and positioned on a P5002A multi‐electrode array system (Alpha MED Scientific Inc., Osaka, Japan). To secure contact between the slice and electrodes and to improve mechanical stability, a piece of nylon mesh and a slice anchor harp were placed on top of the slice. The slice was maneuvered to position the hippocampus on the array. The chamber was perfused with oxygenated ACSF at a rate of 2 mL/min at 32°C. After the slice had settled in the recording chamber, field excitatory postsynaptic potentials (fEPSPs) were generated in the CA1 region of the hippocampus by stimulating downstream electrodes in the CA1 and CA3 regions of the hippocampus along the Schaffer collateral pathway. Input/output curves (I/O curves) were generated by applying increasing stimulus currents to the pathway from 5 μA to 100 μA and recording the responses. The threshold stimulus for generating fEPSPs was determined as 30%–40% of the stimulus strength needed to generate the maximum fEPSP amplitude during the I/O curve measurement. The slice was stimulated once every 30 s until a stable baseline lasting at least 10 min was observed. Long‐term potentiation (LTP) was induced using 100 high‐frequency stimulation pulses at 100 Hz applied three times with 30‐s intervals. Next, baseline stimulation was resumed, and fEPSPs were recorded for at least 60 more min. For all recordings, we used the MED‐64 system and Mobius software (Alpha MED Scientific Inc). Potentiation was calculated as the percent increase of the mean fEPSP descending slope (10–90 section) after high‐frequency stimulation and normalized to the mean fEPSP descending slope of baseline recordings during 3 min prior to tetanus.

### 2.12 Statistical Analysis

All data are presented as means ± standard error of the mean (SEM). GraphPad Prism 9 Software (La Jolla, CA, USA) was employed for statistical analyses. Comparisons between groups of experimental results were conducted using one‐way ANOVA with Fisher LSD post hoc test or an equivalent non‐parametric (Kruskal‐Wallis) or non‐equal distribution (Brown‐Forshyte) tests whichever was applicable based on the sample type and distribution. A minimum of three independent measurements was performed for all data (*n* ≥ 3), and the exact “*n*” animal numbers are specified in the figure legends. Levels of significance were denoted as follows: *p < 0.05, **p < 0.01, ***p < 0.001. Data points impacted by an animal's condition which was not part of the experimental protocol or the low quality of its cranial window were excluded from the study.

## 3 Results

### 3.1 Age‐Related Escalation of Senescent CMVEC Burden in the Mouse Brain

To elucidate the effects of aging on senescence within the brain, we utilized flow cytometry analysis (Figure 1B,C). This investigation involved various age cohorts of p16‐3MR mice, facilitating the measurement of cell senescence levels. To assess endothelial cell senescence, we quantified the prevalence of p16‐RFP+ cells within the CD31+ endothelial cell population, subsequent to the removal of cellular debris and aggregates from the brain‐derived single‐cell suspensions (Figure S1A). Identification of the senescent endothelial cell sub‐population was based on the intensity of the RFP‐Booster signal and the morphological characteristics of CD31+ cells (Figure 1B and Figure S1B–E). Our data reveal a marked age‐related escalation in the proportion of p16‐RFP+/CD31+ senescent endothelial cells in the mouse brain (Figure 1C), highlighting the progressive nature of endothelial senescence with aging. Notably, this increase became statistically significant in middle‐aged mice, starting at the age of 15–17 months, identifying a critical time window where senolytic treatment may be most effective in mitigating neurovascular dysfunction and delaying cognitive decline. To evaluate senescence in other components of the neurovascular unit, we assessed microglial senescence (CD11b+/P16+), astrocyte senescence (GLAST+/p16+ cells), and pericyte senescence (CD140b+/p16+) (Figure S1F–K). Microglial senescence remained low, comprising approximately 1% of all microglia, without a detectable age‐related increase across our time course (up to 24 months of age, Figure S1I). We also observed a limited number of senescent astrocytes, with significant increases only occurring by 27 months of age (Figure S1J). Pericyte senescence increased much more than astrocyte or microglial senescence (as a percentage of total pericytes) but the increase was also not detectable until advanced age (21–27 months). This suggests that while endothelial cells exhibit a progressive accumulation of senescence starting in middle age, astrocytes and pericytes follow a delayed trajectory, with more pronounced changes emerging in very old age. As a result of the early rise in endothelial senescence, we elected to focus most of our experiments on this cell type.

**FIGURE 2:**
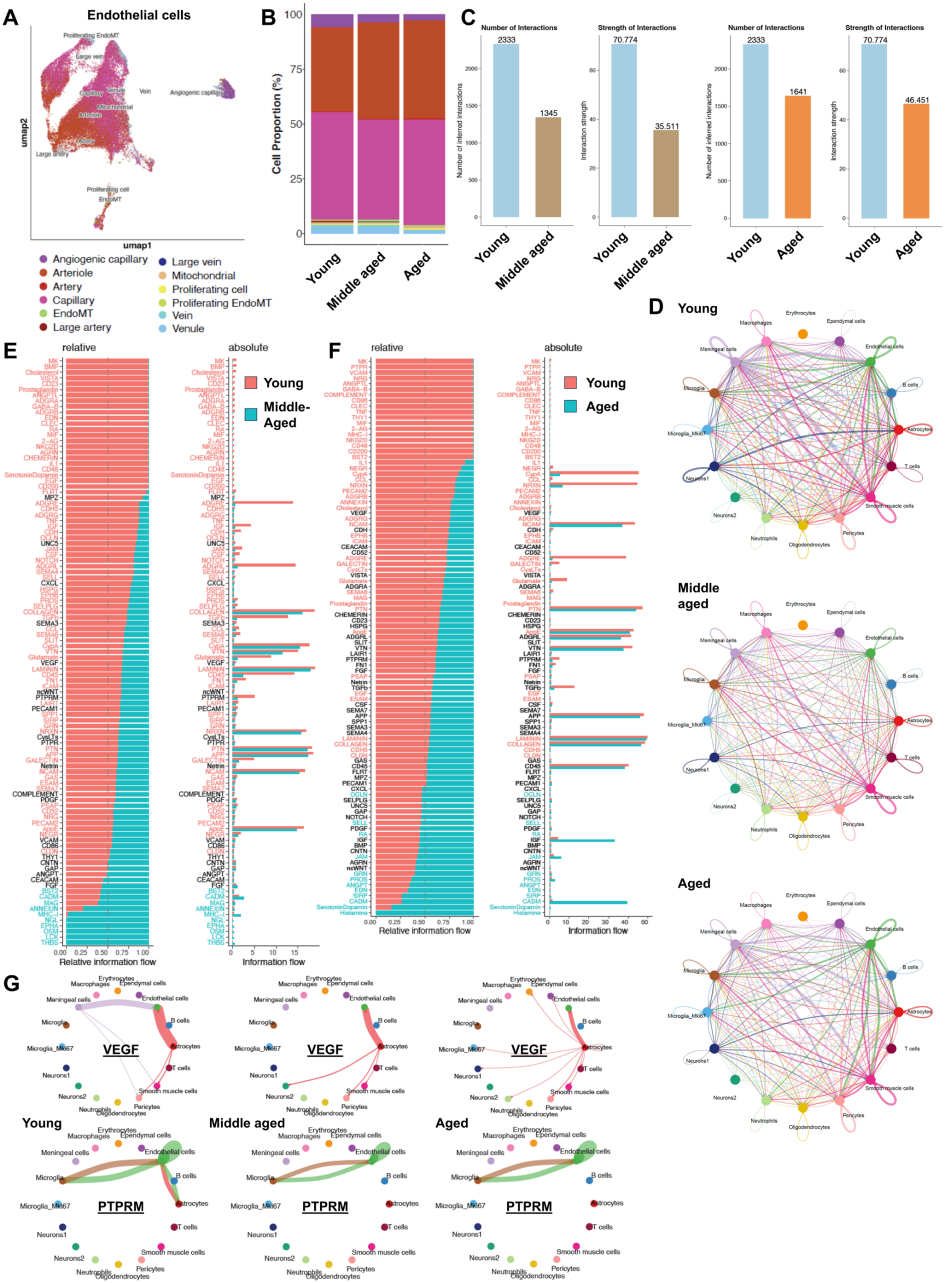
Age‐related alterations in endothelial cell populations, cell–cell interactions, and signaling networks. (A) UMAP plot showing the clustering of endothelial cell subtypes identified across young, middle‐aged, and aged samples. Cell populations are color‐coded and include angiogenic capillary, arterioles, arteries, capillaries, venules, veins, large arteries/veins, mitochondrial‐rich cells, proliferating endothelial cells (Proliferating EndoMT), and cells undergoing endothelial‐to‐mesenchymal transition (EndoMT). Each group is represented by four individual single‐cell data from four individual mice. (B) Bar plot showing the proportions of endothelial cell subtypes across young, middle‐aged, and aged groups, highlighting age‐related shifts. The angiogenic endothelial cell population declines with age, indicating reduced vascular repair capacity. This decrease, especially in aged samples, suggests impaired endothelial turnover, diminished angiogenic responsiveness, and compromised cerebrovascular function. (C) Quantification of the number of intercellular interactions and the strength of these interactions across age groups from the CellChat outcome. Left: Total number of predicted ligand‐receptor interactions decreases in middle aged brain together with the interaction strength (calculated based on cumulative interaction scores) Right: Total number of interaction and interaction strengths are slightly better in aged brains than in middle aged samples, indicating compensatory changes in signaling dynamics. (D) Network diagrams visualizing cell–cell communication patterns in young, middle‐aged, and aged samples. Nodes represent different cell types, and edges represent ligand‐receptor‐mediated interactions, with line thickness proportional to interaction number. (E, F) Relative and absolute information flow of key signaling pathways across age groups. (E) Comparison between young and middle‐aged, and (F) comparison between young and aged groups. Top signaling pathways in the young group with increased (red) or decreased (blue) activity are highlighted, with pathways ranked based on relative information flow. (G) Detailed visualization of VEGF and PTPRM signaling networks in young, middle‐aged, and aged groups. Arcs represent ligand‐receptor interactions between different cell types, with line thickness corresponding to interaction strength. Edge colors represent the source (sender). In young samples, VEGF signaling shows strong, balanced connections with vascular‐related cells, indicating robust vascular support. In middle‐aged samples, reduced interaction strength suggests early vascular communication decline. In aged samples, VEGF signaling is further diminished, implying compromised angiogenesis and vascular dysfunction. PTPRM regulates cell–cell adhesion, signal transduction, and vascular integrity. In young samples, its interactions are strong and widespread, supporting endothelial junctions. Middle‐aged samples show shifted patterns, reflecting changes in adhesion regulation. In aged samples, altered interactions suggest compensatory or dysregulated signaling linked to vascular leakage and dysfunction.

We performed a detailed analysis of scRNA‐seq data from mouse brains of different ages, ensuring cell identities were accurately determined following rigorous quality control checks. By employing unbiased Louvain clustering and identifying cluster‐specific markers through the MAST method, we pinpointed a distinct cluster of endothelial cells. Acknowledging the challenge of dropout events—where genes expressed at low levels may not be fully detected—in single‐cell sequencing, we introduced a modified enrichment score for the senescence marker gene set for each cell, as detailed in the Methods section. This approach benefits from accounting for both the level of expression and the breadth of senescence‐related genes expressed by each cell. Our analysis uncovered a significant subgroup of senescent endothelial cells within the brains of middle‐aged mice (Figure 1D). Endothelial cells display substantial transcriptomic diversity, prompting us to categorize subclusters into arterial, capillary, and venous endothelial cells based on specific, previously recognized markers (Ahire et al. 2023). Our results highlight that senescence is predominantly observed in capillary endothelial cells as mice age (Figure 1D). Consistent with this, further annotation of endothelial cell clusters by subtype (Figure 2A) based on published annotation sets (Walchli et al. 2024) indicates that the angiogenic endothelial cell population declines with age (Figure 2B). These decreases, especially in aged samples, suggest impaired endothelial turnover, diminished angiogenic responsiveness, and compromised cerebrovascular function. These cellular and molecular biology findings from AFCS and scRNA‐seq further reinforce the previously observed functional and structural changes in the cerebrovascular system, highlighting their microvascular rather than macrovascular origin.

### 3.2 Age‐Induced Cell–Cell Interaction Changes in Chronological Aging

Using the CellChat algorithm to test cell–cell interactions, we found a decline in overall cell–cell interactions with aging (Figure 2 and Figure S3). The bar charts on Figure 2C show a significant decline in both the number and strength of cellular interactions as the brain transitions from young to middle‐aged stages. Surprisingly, these parameters show slight improvements in successfully aged mice. The number of interactions decreases from 2333 in young mice to 1345 in middle‐aged mice and increases slightly to 1641 in aged mice. Similarly, the strength of interactions declines sharply, indicating weakened communication among brain cells, which is crucial for maintaining homeostasis and cognitive function.

The network interaction maps representing cellular communication between different cell types (Figure 2D) are visualizing cell–cell communication patterns in young, middle‐aged, and aged samples. Nodes represent different cell types, and edges represent ligand‐receptor‐mediated interactions, with line thickness proportional to interaction strength. The analysis further details the age‐dependent changes in cerebrovascular cellular interactions, emphasizing alterations in microvascular function, immune response, and neurovascular coupling. The strong connectivity observed in endothelial cells, pericytes, and smooth muscle cells suggests that microvascular dysfunction emerges as a key factor in aging‐related cerebrovascular decline. A reduction in endothelial‐immune and endothelial‐neuronal interactions with age may indicate impaired angiogenesis, diminished vascular plasticity, and endothelial senescence, contributing to cerebrovascular dysfunction (Figure S3A–C). Additionally, age‐related changes in immune cell connectivity, particularly in microglia and macrophages, suggest an increasing role of neuroinflammation, potentially due to BBB dysfunction and heightened susceptibility to neurodegenerative processes. The decline in neuronal‐vascular interactions with age further supports the hypothesis that aging disrupts neurovascular coupling, reducing cerebral blood flow and impairing long‐term potentiation (Figure S2) and cognitive function (Figure 6). Notably, astrocytes remain strongly interconnected, reinforcing their regulatory role in maintaining homeostasis, though their ability to compensate for age‐related vascular deficits may be limited.

The heatmaps comparing middle‐aged versus young and aged vs. young samples also show a differential loss of interactions, with endothelial cells among the most affected cell types (Figure S3A). The scatter plots indicate that endothelial cells in young brains play a central role in neurovascular communication, but their interaction strength declines significantly in middle‐aged and aged brains (Figure S3C). This suggests that endothelial cells are undergoing senescence earlier than other cell types, which might contribute to a progressive decline in neurovascular functions and interactions. The loss of endothelial cell interactions might lead to BBB dysfunction, reduced microvascular density, and impaired neurovascular coupling (Figure S3F,G).

The Figure 2E,F presents comparative analyses of signaling interactions and information flow between different age groups: (Middle‐Aged vs. Young) (Figure 2E) and Aged vs. Young (Figure 2F). The relative and absolute information flow metrics indicate age‐related alterations in intercellular communication networks, particularly in neurovascular and immune signaling. In middle‐aged and aged mice, there is a reduction in information flow for key vascular and neuroprotective factors (e.g., vascular endothelial growth factor (VEGF), neurogenic locus notch homolog protein (NOTCH), LAMININ, and platelet‐derived growth factor (PDGF)), which are crucial for vascular integrity, neurovascular coupling, and BBB maintenance. The decline is more pronounced in aged mice, suggesting a progressive breakdown of vascular support mechanisms. A strong upregulation of inflammatory and immune‐modulatory factors (e.g., tumor necrosis factor α (TNF‐α), interleukin (IL)‐6, C‐X‐C motif chemokine ligand (CXCL), and complement system proteins) is observed in both middle‐aged and aged mice. This suggests that with aging, chronic low‐grade inflammation (“inflammaging”) intensifies, contributing to endothelial dysfunction, increased BBB permeability, and neurodegenerative processes. Comparing middle‐aged versus young (Figure 2E), we observe an early reduction in vascular‐supporting signals and a concurrent increase in inflammatory signaling. By the aged stage (Figure 2F), this shift becomes even more extreme, reinforcing that the middle‐aged state is a critical window for vascular health interventions. Signals related to serotonin, dopamine, and histamine are altered, especially in the aged group, which could indicate age‐related disruptions in neurovascular and neuromodulatory function. These changes may contribute to cognitive decline, mood disorders, and impaired blood flow regulation in aging brains.

A comprehensive analysis of VEGF and Protein Tyrosine Phosphatase, Receptor Type M (PTPRM) signaling networks across young, middle‐aged, and aged groups was performed (Figure 2G). Arcs represent ligand‐receptor interactions between different cell types, with line thickness indicating interaction strength and edge colors denoting the signaling source (sender). In young samples, VEGF signaling exhibits strong and well‐balanced interactions among vascular‐associated cells, reflecting robust endothelial responsiveness and effective vascular support. In middle‐aged samples, the reduced interaction strength suggests an early decline in vascular communication, potentially signaling the onset of angiogenic dysfunction. In aged samples, VEGF signaling is further diminished, implying compromised angiogenesis and vascular dysfunction. PTPRM, is a receptor‐type protein tyrosine phosphatase that plays a critical role in cell adhesion, signaling, and regulation of cellular interactions. It is a transmembrane protein that functions in cell–cell communication, particularly in neuronal and vascular systems. In young samples, PTPRM interactions are strong and widely distributed, supporting endothelial junction stability and vascular integrity. In middle‐aged samples, changes in interaction patterns suggest a transition in adhesion regulation, potentially reflecting compensatory mechanisms for early vascular decline. By old age, PTPRM signaling is significantly altered, indicative of compensatory or dysregulated adhesion signaling, potentially contributing to vascular leakage and dysfunction. These findings suggest that while VEGF signaling weakens due to impaired endothelial sensitivity, concurrent PTPRM dysregulation may exacerbate age‐related vascular instability.

### 3.3 Senolytic Therapy Mitigates Age‐Related Decline in NVC Responses

**FIGURE 3:**
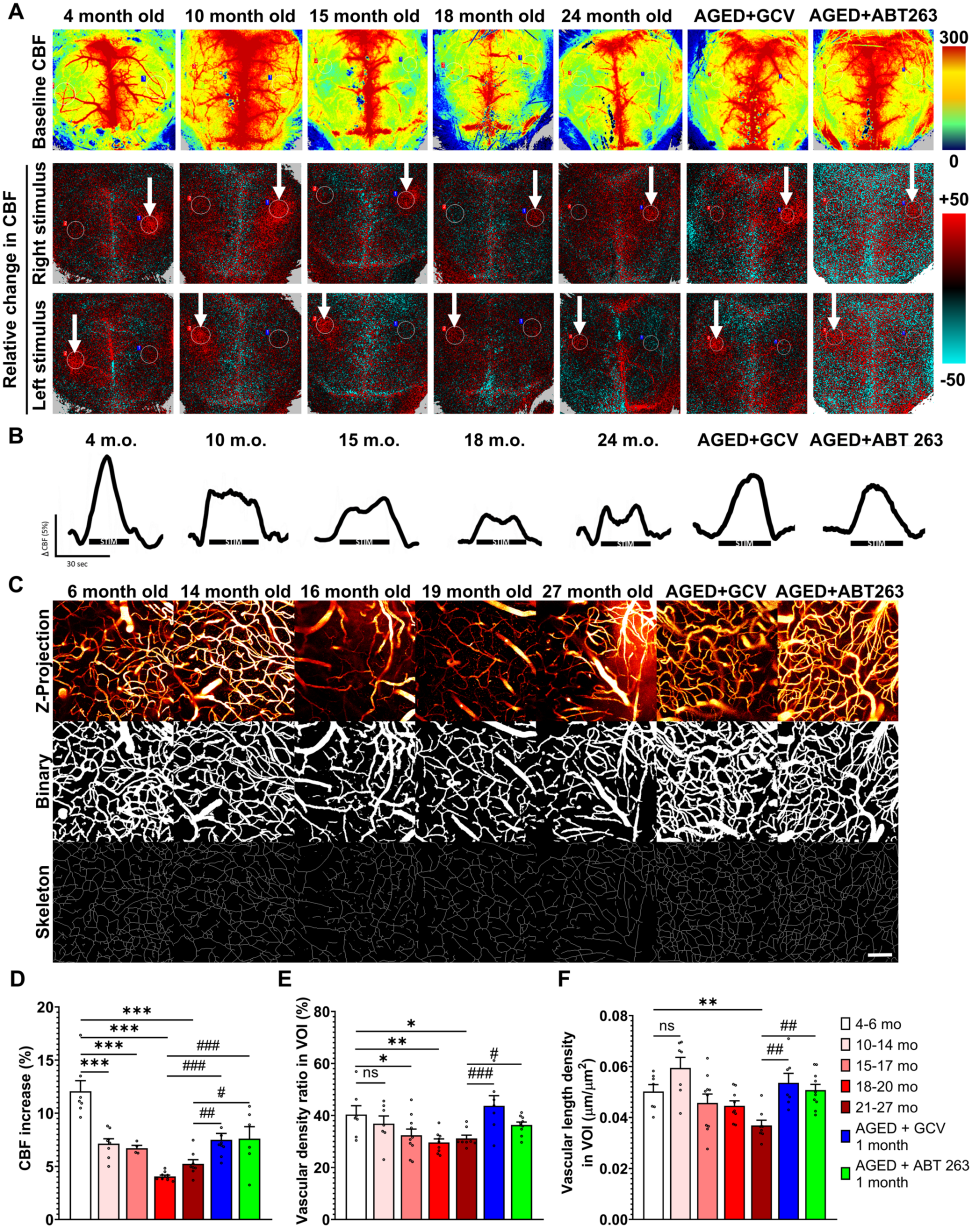
Senolytic therapy mitigates age‐related decline in NVC responses and restores microvascular density in the aged mouse cortex. (A) Representative pseudocolour laser speckle flowmetry maps of baseline CBF (upper row; shown for orientation purposes) and CBF changes in the whisker barrel field relative to baseline during contralateral whisker stimulation (middle and bottom row, right and left oval, 30 s, 5 Hz) in mice of different ages and aged mice (~20‐21‐month‐old) that received GCV or ABT263 previously at 18 month of age. Color bar represents CBF as percentage change from baseline. (B) Shows the time‐course of CBF changes after the start of contralateral whisker stimulation (horizontal bars). Summary data are shown in panel (D)). Data are mean ± SEM. (*n* = 6–8 in each group), *p < 0.05, **p < 0.01, ***p < 0.001 versus Young (4–6 months); #*p* < 0.05, ##*p* < 0.01 versus Aged (18–20, or 21–27 months). (one‐way ANOVA with post hoc Tukey's tests). (C) Segmentation of blood vessels on two‐photon microscopy images. Original z‐stack images (first row) obtained from the brains from mice of different ages and aged mice (~20–21 month old) that received GCV or ABT263 were processed using a modified image analysis macro (Nyul‐Toth et al. 2021) to generate binary vascular images (second row). Skeleton images were used to calculate vascular length density. Scale bar: 100 μm. (E) Vascular coverage, represented as the percentage of the area of the maximal intensity projection of the volume of interest (VOI). (F) Vascular length density, measured as the length of the vascular skeleton in micrometers per μm^2^ of the maximal intensity projection of the VOI. Data are mean ± SEM. *p < 0.05, **p < 0.01, versus Young; * #*p* < 0.05, ##*p* < 0.01, ###*p* < 0.001 versus Aged. (*n* ≥ 7) with one‐way ANOVA followed by Fisher LSD post hoc test.

To explore the alterations in NVC responses associated with aging, we evaluated functional hyperemia within the whisker barrel cortex across various age groups of mice (Figure 3). CBF responses, induced by contralateral whisker stimulation, exhibited a significant decline in older mice when compared to younger controls, indicating a deterioration in NVC. Representative laser speckle contrast images and CBF tracings are shown in Figure 3A,B, summary data are shown in Figure 3D.

To investigate the impact of cellular senescence on neurovascular dysfunction, we assessed NVC responses in aged mice following senolytic treatment with either GCV or ABT263, administered for 1 month at 17–18 months of age—a previously determined critical window when senescent endothelial cells begin to accumulate—and evaluated the effects 1 month post‐treatment at 19–20 months of age. While these two senolytics target senescent cells globally, the timing of our delivery follows significant accumulation of senescent endothelial cells and precedes significant accumulation of senescence in other cell types in the brain, suggesting endothelial cells are the primary cell type affected by the senolytics. Remarkably, in aged mice, these senolytic interventions significantly enhanced NVC responses, effectively moving them towards levels detected in young control animals (Figure 3A,B,D). Our scRNA‐seq CellChat analysis shows that the overall neurovascular network structure appears similar, but there are subtle differences in the strength of connections between cell types of the neurovascular unit (Figure 2D). In the middle‐aged brain, some connections appear less pronounced, suggesting a weakening of interactions. Particularly, the reduced connection among endothelial cells, neurons, and supporting glial cells suggests early signs of neurovascular dysfunction. This aligns with known age‐related declines in neurovascular coupling efficiency, which contribute to cognitive impairment over time. Microglia, key immune cells of the brain, maintain strong connectivity, but alterations in their interactions with endothelial cells and neurons (Figure 2D) indicate increased neuroinflammation and endothelial dysfunction, both of which are characteristic of aging‐related neurovascular decline (Figure 6F).

### 3.4 Senolytic Therapy Restores Microvascular Density in the Aged Mouse Cortex

Building on prior research indicating that aging leads to substantial microvascular rarefaction in the brain, thereby contributing to cognitive decline, we analyzed changes in microvascular densities within the mouse cortex (Nyul‐Toth et al. 2021; Tucsek et al. 2014). Using two‐photon microscopy, we adopted a proven image processing and analysis technique to examine cortical vascularization (Nyul‐Toth et al. 2021; Ahire et al. 2023; Gulej et al. 2023b). Specifically, z‐stack images captured from the WGA‐A594 channel were subjected to dimension reduction via maximum intensity projection. This step was followed by a machine learning‐based segmentation to delineate the vasculature accurately, alongside the application of filters to remove noise and outliers. The processed images were converted into binary format for quantification purposes (Figure 3C). To minimize the impact of larger blood vessels within the analysis field, a skeletonization technique was employed, enhancing the accuracy of our vascular density measurements by ensuring they were unaffected by vessel size variations within the VOI. Our findings indicate a notable decrease in vascular density in the cortex with age when compared to the young control group (Figure 3E,F). Remarkably, in aged mice treated with either ABT263 or GCV senolytic, these interventions significantly increased microvascular density (Figure 3C,E,F).

These results were further substantiated by our scRNA‐seq data. By further clustering the endothelial cells, we identified distinct functional endothelial subtypes, including angiogenic endothelial cells, which play a critical role in vascular remodeling and repair. Notably, the proportion of angiogenic endothelial cells declines with aging, supporting the observed reduction in vascular density in the aging brain (Figure 2A,B and Figure S3F). This finding aligns with the progressive loss of cerebromicrovascular density, which contributes to impaired cerebral blood flow in older individuals.

Additionally, we observed an increase in Endothelial‐to‐Mesenchymal Transition (EndoMT) cells with aging (Figure 2A,B), which is strongly linked to endothelial senescence and the SASP. Senescent endothelial cells release pro‐inflammatory cytokines, chemokines, growth factors, and matrix‐remodeling enzymes, including IL‐6, IL‐1β, transforming growth factor β (TGF‐β), and MMPs, which create a chronic inflammatory microenvironment. These SASP factors can act as key drivers of EndoMT, promoting the transition of endothelial cells into mesenchymal‐like cells, leading to vascular fibrosis, stiffening, and maladaptive remodeling in large vessels and increased vascular fragility in the microvasculature. The increased presence of EndoMT in aged brains suggests that senescent endothelial cells actively contribute to vascular pathology. This chronic inflammatory state may accelerate vascular cognitive impairment, highlighting the importance of targeting senescent cells to mitigate EndoMT‐driven vascular deterioration.

### 3.5 Enhancement of BBB Function in Aged Mice Through Senolytic Therapy

**FIGURE 4:**
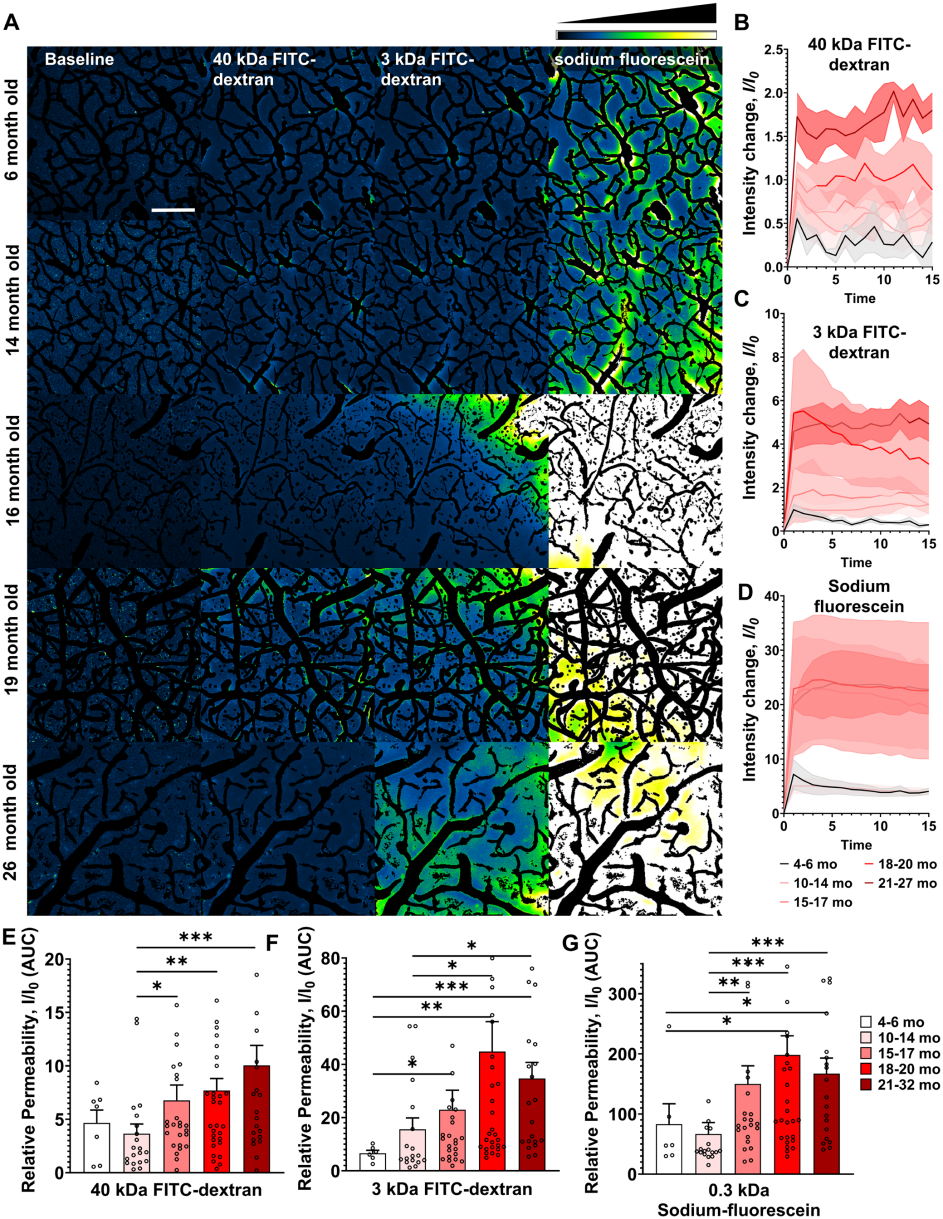
Age‐related disruption of the blood–brain barrier (BBB) in the mouse brain cortex. (A) Assessment of cerebromicrovascular permeability to fluorescent, FITC‐labeled tracers using two‐photon imaging in brains of mice of different ages. Following retroorbital injection of tracers with differing molecular weights, changes in tracer fluorescence intensity within the extravascular space and brain parenchyma were examined. Maximum projection images (z‐stack) captured post‐injection were further processed by subtracting images of segmented cerebral microvasculature to highlight tracer extravasation. Intensity plots, based on average projections from time‐stacks, reveal an increase in tracer leakage across the BBB in middle‐aged and aged mice compared to younger controls. The fluorescence intensity scale is provided in the top right corner. Scale bar: 100 μm. (B–D) Fluorescent intensity curves over the baseline from different aged groups. The quantification of the Area Under the Curve (AUC) reflects the relative permeability of the BBB. Panel E‐G: Summary data for cerebromicrovascular permeability to fluorescent tracers with different molecular weights (E: 40 kDa; F: 3 kDa; G: 0.3 kDa) in mice of different ages. Data are mean ± SEM. *p < 0.05, **p < 0.01, (*n* > 10 for all groups) with Kruskal‐Wallis test (non‐parametric ANOVA).

Using intravital two‐photon microscopy, the relative permeability of the fluorescent tracers was measured. Tracers with different molecular weights were injected retro‐orbitally in a decreasing order. The permeability of each tracer was monitored for 15 min. Vascular masks were segmented on the z‐stack projections with supervised machine learning and subtracted from the images to exclusively measure changes in the relative intensity of the extravasated tracers. Figure 4A illustrates the changes in background‐corrected fluorescent intensity over time within the brain parenchyma of mice across various experimental age groups, following the introduction of tracers of different sizes. For quantification purposes, the area under the curve of the normalized intensity changes over time was calculated for each tracer, serving as a measure of relative BBB permeability (Figure 4B–D). In both young and aged animals, this metric of relative BBB permeability aligned with our previously published data for all three tracers (Nyul‐Toth et al. 2021). We observed a progressive increase in relative BBB permeability with age for each of the tracers assessed (Figure 4E–G), thereby expanding upon our earlier results (Nyul‐Toth et al. 2021).

**FIGURE 5:**
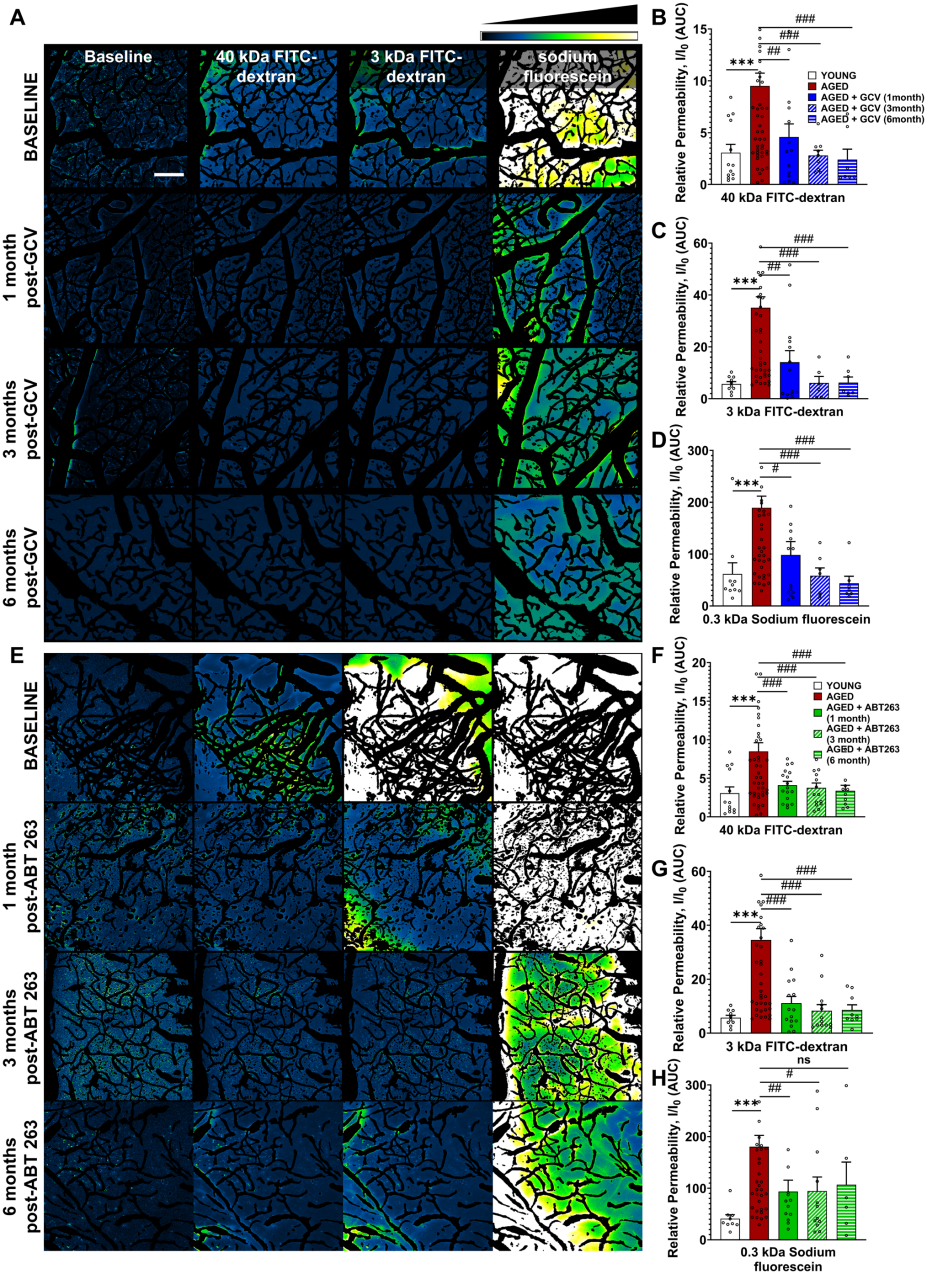
Enhancement of BBB function in aged mice through senolytic therapy. Two‐photon‐imaging‐based measurement of microvascular permeability to fluorescent tracers in brains of young control and aged p16‐3MR mice that received vehicle, ganciclovir (GCV) or ABT263. (A) Display of representative intensity maps showcasing the mouse brain vasculature during microvascular permeability assessments. Treatment with GCV notably diminishes extravascular tracer intensities in maximum projection images, indicating a reduction in vascular permeability. The scale bar represents 100 μm. (B–D) These panels present the quantification of relative permeability changes in aged mice treated with GCV. In aged mice, senolytic treatment with GCV effectively decreased BBB permeability for all tracers tested one‐month post‐treatment (B: 40 kDa; C: 3 kDa; D: 0.3 kDa), aligning it more closely with the permeability levels observed in young control mice. Further assessments of BBB permeability at 3 and 6 months after treatment showed no significant increases in permeability levels, demonstrating the long‐term effectiveness of the treatment in preserving the improved integrity of the BBB. (E) Representative intensity maps illustrating mouse brain vasculature during microvascular permeability assessments before and after treatment with ABT263. This pharmacological approach to senolysis effectively reduces extravascular tracer intensities in maximum projection images, implying enhanced barrier functions. Scale bar: 100 μm. (F–H) Quantification of relative permeability changes in aged mice treated with ABT263. In aged mice, senolytic treatment with ABT263 resulted in a substantial reduction in BBB permeability to all tracers tested one‐month post‐treatment (F: 40 kDa; G: 3 kDa; H: 0.3 kDa), aligning it more closely with the permeability levels observed in young control mice. Follow‐up assessments of BBB permeability at 3 and 6 months after the treatment showed stable BBB permeability levels, underscoring the lasting impact of the treatment in preserving the improved integrity of the BBB over an extended period. Data are mean ± SEM. (*n* > 10 for each group) ***p < 0.001 versus Young; #*p* < 0.05, ##*p* < 0.01, ###*p* < 0.001 versus Aged with Kruskal‐Wallis test.

To investigate the impact of cellular senescence on BBB disruption, we evaluated the efficacy of senolytic therapies on age‐related increases in BBB permeability. In aged mice starting to be administered at 18 months of age, senolytic treatments with both GCV (Figure 5A–D) and ABT263 (Figure 5E–H) successfully decreased BBB permeability for all tracers tested, bringing it closer to the levels seen in young control mice. We further assessed whether BBB permeability would return to levels seen in untreated aged animals over time after the senolytic treatment. Measurements of BBB permeability at 3‐ and 6‐months post‐treatment indicated no additional increases, implying that a single round of senolytic therapy yields long‐lasting benefits (Figure 5A–H).

The heatmap in the provided figure (Figure S3G) highlights gene ontology biological processes (GOBP) related to BBB function and cellular senescence, with enrichment scores reflecting their activity across young, middle‐aged, and aged mice. Processes related to the maintenance and establishment of the BBB (e.g., GOBP‐MAINTENANCE‐OF‐BLOOD‐BRAIN‐BARRIER and GOBP‐ESTABLISHMENT‐OF‐BLOOD–BRAIN‐BARRIER) show a notable decrease in enrichment (darker colors), suggesting age‐related impairment in BBB stability. The negative regulation of blood–brain barrier permeability is also reduced, which could imply increased BBB permeability, a hallmark of neurovascular aging and neurodegenerative susceptibility. The presence of stress‐induced premature senescence implies that endothelial cells in the BBB are experiencing oxidative and inflammatory stress, accelerating their dysfunction. Processes related to lipid and xenobiotic transport across the BBB (GOBP‐LIPID‐TRANSPORT‐ACROSS‐BLOOD‐BRAIN‐BARRIER and GOBP‐XENOBIOTIC‐TRANSPORT‐ACROSS‐BLOOD–BRAIN‐BARRIER) show altered enrichment, indicating potential disruptions in nutrient exchange and fluid balance in the aging brain.

### 3.6 Effects of Senolytic Treatments on Cognitive Function in Aged Mice

**FIGURE 6:**
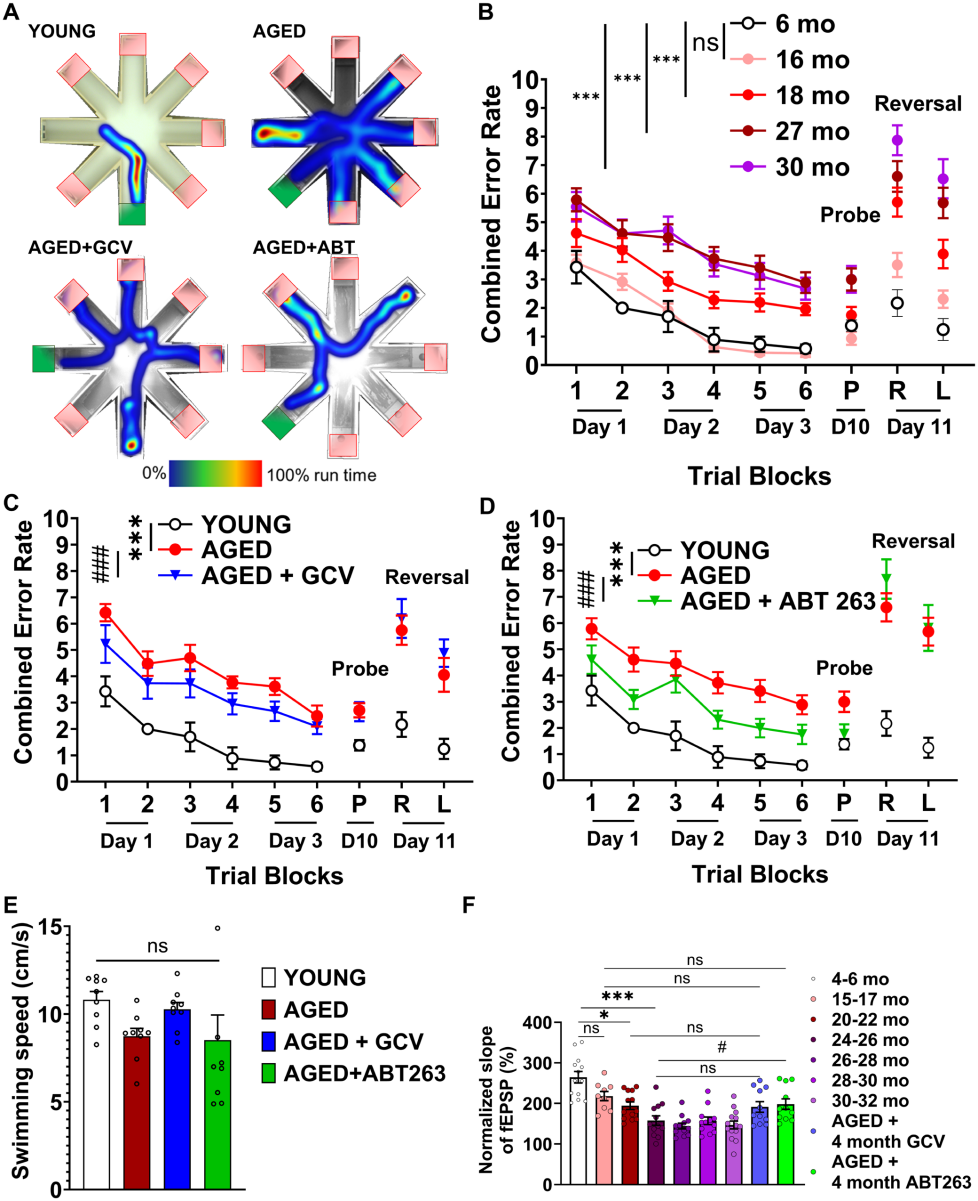
Impact of senolytic treatments on cognitive functions in aged mice. p16‐3MR mice of different ages were subjected to vehicle, ganciclovir (GCV), or ABT263 treatments and assessed for their spatial learning and memory using the Radial Arm Water Maze (RAWM). (A) Heatmap illustrates the percentage of time spent in different maze locations on probe day 10 (“P”) for a randomly selected animal from each group. Notably, the untreated aged mice spent more time and traversed a longer path to locate the hidden escape platform, frequently revisiting previously entered arms and accumulating working memory errors, in contrast to the young mice. (B) Chronological aging impairs learning and memory functions in mice. Advanced age effected cognition in mice starting from 18 month to learn and from 16 months to relearn the task. During the learning phase (days 1 to 3) and on retrieval day 10, aged mice displayed higher combined error rates compared to young mice. (C, D) Treatment with GCV (Panel C) or ABT263 (Panel D) significantly enhanced learning performance in aged mice relative to their untreated counterparts. Additionally, older mice committed more errors than young mice during the reversal trial, with no noticeable improvement from senolytic treatments during this phase. The combined error rate was computed by adding one error for each incorrect arm entry plus an error for every 15 s of inactivity. Data are mean ± SEM (*n* = 10–15 per group). Statistical significance indicated by ***p < 0.001 versus Young; ###*p* < 0.001 versus Aged using Repeated Measure ANOVA, demonstrating the beneficial effects of senolytic treatments on enhancing cognitive functions in aged mice, particularly in spatial learning, but less so in tasks requiring cognitive flexibility. (E) Average swimming speeds in the RAWM for each group, analyzed with One‐way ANOVA, revealed no significant differences across treatments, underscoring that swimming speed did not influence learning performance. Data are presented as mean ± SEM (*n* = 10–15 per group). (F) Age‐related vascular senescence does not alter the long‐term potentiation (LTP) in the hippocampal synapses significantly until late age. LTP was induced in mice of different ages and in mice treated with senolytics (*n* ≥ 12 slices from 5 to 6 mice in each group). The graph depicts fEPSP slope values normalized to the baseline response and expressed as percent values (%), 60 min post‐induction of LTP (Data shown as mean ± SEM. ***p < 0.001 versus Young; #*p* < 0.05 versus Aged; *p* < 0.05, Mann Whitney test).

To evaluate the impact of aging on learning and memory, we utilized the RAWM test, a well‐established method for assessing spatial learning and memory in mice. We monitored changes in the frequency of arm entrance errors from session to session as an indicator of learning performance. Errors were quantified by combining two metrics: one error for each incorrect arm entry and an additional error for every 15 s spent without exploring the arms. This combined error rate served as a comprehensive indicator of the mice's learning ability over time. Our analysis revealed distinct differences in the learning curves between mice of different ages, aligning with the hypothesis that aging is associated with impairments in spatial learning ability (Figure 6A,B). Intriguingly, senolytic treatments at 18 months of age with both GCV and ABT263 significantly enhanced learning performance in aged mice, as evidenced in Figure 6A,C,D. However, when examining aspects of memory retention and reversal learning—where the escape platform's location is altered to assess the animal's adaptability to new spatial information—senolytic treatments did not manifest a significant impact. This finding suggests that while senolytic interventions can ameliorate certain aspects of cognitive decline related to aging, they may not influence all facets of cognitive flexibility equally. Additionally, it was observed that senolytic treatments did not alter swimming speed (Figure 6E), indicating that the improvements in learning performance were not attributable to changes in locomotor activity.

Chronological aging could reduce functional hyperemia by impairing neuronal activation. To examine this possibility, we measured evoked neuronal activation by assessing field excitatory postsynaptic potentials (fEPSP) in hippocampal preparations from animals from different age groups; long‐term potentiation (LTP) was successfully induced in aged and in senolytic treated mice, similar to the LTP in young mice. Chronological aging did not affect neuronal function and synaptic plasticity (Figure 6F) until late elderly age, and senolytic treatments resulted in improved LTP (Figure S2A). Notably, synaptic plasticity started significantly decreasing starting from 24 months of age, which is a much later timepoint compared to the start of senescence, endothelial cell accumulation, and BBB breakdown. Therefore, chronological aging is unlikely to contribute to impaired functional hyperemia by modulating neuronal activation.

## 4 Discussion

The pivotal finding of our study is that endothelial senescence is the primary driver of neurovascular dysfunction in aging. Senescence endothelial markers appear as early as middle age, whereas other brain cell types exhibit senescence predominantly in late life. This identifies middle age as a critical window for intervention before neurovascular dysfunction becomes irreversible. Importantly, our study demonstrates that the targeted depletion of senescent microvascular endothelial cells (CMVECs) significantly enhances NVC responses, augments brain capillarization, and partially mitigates the chronic increase in BBB permeability. Given that endothelial senescence emerges earlier than in other brain cells, this time window presents a crucial opportunity for targeted senolytic therapies to restore vascular function and prevent the progression of cognitive decline in aging brains.

Our findings demonstrate a time‐dependent increase in senescent endothelial cells within the brain as a result of aging, building upon the results of our previous studies (Kiss et al. 2020a; Ximerakis et al. 2019). Notably, age‐related senescence predominantly affects microvascular rather than macrovascular endothelial cells, underscoring the critical role of senescent cell accumulation within the neurovascular unit (Kiss et al. 2020a). The unexpected early rise in endothelial senescence compared to other brain cell types aligns with the well‐documented early vascular functional and structural changes, such as impaired neurovascular coupling, reduced capillary density, and increased BBB permeability, which precede neuronal dysfunction. This suggests that vascular aging is a primary driver of neurodegenerative processes, rather than a secondary consequence. The observation that neuronal dysfunction occurs later in life, which is supported by our long‐term potentiation measurements that show changes in neuronal function in the oldest age groups, further strengthens the concept of vascular‐driven brain aging. This evidence highlights the therapeutic potential of targeted senolytic interventions, specifically aimed at eliminating senescent endothelial cells, as a strategy to preserve neurovascular integrity, protect neuronal health, and mitigate cognitive decline.

The clinical significance of our research is further supported by recent findings showing that in aged human brain tissues, there is an upregulation of senescence markers in endothelial cells and other cell types of the neurovascular unit (Xu et al. 2022; Dehkordi et al. 2021). This alignment between our animal model findings and human clinical data highlights the translational potential of our research and suggests that early interventions targeting vascular senescence may offer a viable strategy to delay or prevent age‐related cognitive impairment and neurodegenerative diseases.

The pathophysiological impact of CMVEC senescence induced by aging is complex. Crucially, CMVECs play a pivotal role in mediating NVC responses (Tarantini et al. 2019; Toth et al. 2015c; Chen et al. 2014). NVC, also known as functional hyperemia, is a fundamental homeostatic mechanism that ensures blood supply is synchronized with local neuronal activity demands (Toth et al. 2017). The results of our study are in alignment with findings from several prior investigations, confirming that aging in the mouse brain is associated with a progressive impairment of NVC responses (Toth et al. 2017; Tarantini et al. 2021; Csiszar et al. 2019; Gulej et al. 2023c). Importantly, evidence from various studies also underscores that NVC impairment is a critical aspect of human aging (Csipo et al. 2019; Jor'dan et al. 2020; Lipecz et al. 2019; Iadecola 2017). This cross‐species observation highlights the universal nature of neurovascular aging and its implications for brain health across the lifespan. The pathophysiological significance of impaired NVC responses in the aging brain cannot be overstated. NVC impairment has been causally linked to age‐related declines in cognitive performance across species, highlighting a critical mechanism by which neurovascular health directly impacts brain function and overall cognitive integrity (Tarantini et al. 2021; Iadecola 2017). In humans, deteriorations in NVC responses have been associated with various cognitive deficits, including reduced processing speed, memory, and executive function, which are hallmark signs of brain aging (Iadecola 2017). Similarly, in murine models, impaired NVC has been shown to precede neuronal loss, suggesting that targeting neurovascular health may represent a viable therapeutic strategy to mitigate age‐related cognitive impairment (Toth et al. 2017; Tarantini et al. 2021). Our current and prior research underscores the efficacy of senolytic treatments in ameliorating endothelium‐mediated NVC responses in the aged mouse brain, indicating that the accumulation of senescent CMVECs plays a pivotal role in the age‐related dysregulation of CBF (Tarantini et al. 2021). Utilizing the 3‐MR mouse model as a senescence reporter, we have validated the effective clearance of senescent CMVECs via GCV administration and ABT263/Navitoclax treatment (Ahire et al. 2023; Yabluchanksiy et al. 2020). Potential mechanisms through which senescent endothelial cells within the cerebromicrovascular network may adversely impact NVC responses include the disruption of conducted vasodilation, harmful paracrine effects impacting endothelial integrity, the non‐cell autonomous spread of cellular senescence, and consequent endothelial dysfunction. The significance of senescent cells in compromising NVC is further highlighted by recent discoveries showing that ABT263 treatment not only mitigates senescence but also restores functional hyperemia in mice subjected to whole‐brain irradiation (Yabluchanksiy et al. 2020) or chemotherapy (Ahire et al. 2023).

Our scRNA‐seq analysis of cell–cell interactions provides compelling evidence supporting the detrimental role of senescent endothelial cells in neurovascular dysfunction. The data reveal a progressive decline in endothelial cell communication with pericytes and astrocytes, two critical components of the neurovascular unit that regulate NVC and BBB integrity. In middle‐aged and aged mice, we observed a significant reduction in pro‐homeostatic endothelial signaling, including pathways related to nitric oxide (NO) production, VEGF‐mediated angiogenesis, and NOTCH signaling, which are essential for coordinated vasodilation and vascular plasticity. Concomitantly, there is an enrichment of inflammatory and SASP‐related interactions, including increased expression of IL‐6, CXCL, and TGF‐β signaling between senescent endothelial cells and surrounding glial cells. These findings support the hypothesis that senescent endothelial cells propagate cellular dysfunction through harmful paracrine signaling, leading to the non‐cell‐autonomous spread of senescence and exacerbating endothelial dysfunction. Furthermore, our data suggest that senescent endothelial cells exhibit dysregulated connexin and gap junction signaling, which may impair conducted vasodilation, a key mechanism for maintaining NVC responses. This molecular evidence aligns with previous findings that ABT263 treatment not only reduces endothelial senescence but also restores functional hyperemia, reinforcing the concept that targeted senolytic therapy may be an effective strategy to preserve neurovascular function and prevent cognitive decline in aging and disease conditions.

Beyond the impairment of NVC responses, aging is also implicated in promoting microvascular rarefaction in critical regions such as the cortex and hippocampus, contributing to diminished regional CBF and cognitive decline (Nyul‐Toth et al. 2021). Our study is pioneering in charting the progression of age‐related microvascular rarefaction. Prior research has elucidated that brain capillarization is governed by the dynamic interplay between angiogenesis and capillary regression (Ungvari et al. 2018). Within this context, senescent CMVECs exhibit hallmark features such as cell cycle arrest and a marked reduction in angiogenic capabilities, implicating them in the process of microvascular rarefaction (Ungvari et al. 2013). The observed increase in capillary density within the cortex following senolytic treatments significantly reinforces this hypothesis (Ahire et al. 2023). Such findings highlight the detrimental impact of CMVEC senescence on the microvascular landscape and underscore the potential of senolytic interventions to counteract age‐related declines in cerebral capillary networks.

Our scRNA‐seq data reveal a significant decline in angiogenic endothelial cells, marked by reduced expression of VEGF‐A, angiopoietin‐2 (ANGPT2), and delta‐like ligand 4 (DLL4) in middle‐aged and aged mice compared to young brains. This reduction likely contributes to decreased capillary sprouting and vessel maintenance, leading to microvascular rarefaction. Alongside this decline, we observe an increase in anti‐angiogenic and senescence‐associated signaling, including thrombospondin (THBS) 1 and 2, known inhibitors of angiogenesis that impair endothelial proliferation and vessel remodeling. Additionally, TGF‐β/suppressor of mothers against decapentaplegic (SMAD) signaling (elevated TGF‐β2 and SMAD3 expression) is upregulated, promoting vascular dysfunction and fibrosis. Moreover, there is an increased presence of mesenchymal‐like endothelial cells undergoing EndoMT, as indicated by upregulated Snail, Slug, and Twist, suggesting that instead of forming new capillaries, endothelial cells are transitioning into a dysfunctional fibrotic phenotype. Further supporting vascular deterioration, we detect a loss of pro‐survival and vascular maintenance signaling, characterized by reduced expression of VEGF receptor 2, tyrosine kinase with immunoglobulin and epidermal growth factor homology domains 2 (Tie2), and Notch1, which are essential for capillary stability and endothelial survival. Additionally, wingless‐related integration site (Wnt)/β‐catenin signaling, crucial for maintaining BBB integrity, is downregulated, further exacerbating vascular fragility. This dysfunction is coupled with an increase in apoptotic and senescence pathways, as evidenced by elevated p21 (cyclin‐dependent kinase inhibitor (CDKN) 1A) and p16 (CDKN2A) expression in endothelial cells, reinforcing the accumulation of senescent endothelial cells. The upregulation of caspase‐dependent apoptotic markers (caspase‐3 and caspase‐8) in endothelial populations suggests increased endothelial cell death, contributing to progressive vascular regression. These data strongly support the hypothesis that vascular rarefaction in the middle‐aged brain results from both a reduction in pro‐angiogenic endothelial cells and an increase in anti‐angiogenic and senescence‐associated signaling. The combination of thrombospondin‐driven angiogenic suppression, endothelial cell senescence, and increased EndoMT suggests that the aging neurovascular system is shifting towards progressive vessel loss and dysfunction, preceding full‐blown neurovascular impairment seen in advanced age. These findings emphasize middle age as a critical window for intervention, where senolytics or pro‐angiogenic therapies may counteract vascular decline and support neurovascular health.

CMVECs are critical to the structural and functional integrity of the BBB, which is crucial for sustaining an optimal neural microenvironment and, consequently, normal synaptic activity (Nyul‐Toth et al. 2021; Sweeney et al. 2019). Our research demonstrates that aging leads to a progressive disruption of the BBB, corroborating and extending previous observations in both human subjects and murine models (Nyul‐Toth et al. 2021; Sweeney et al. 2019; Verheggen et al. 2020a, 2020b). This age‐related BBB disruption is directly associated with an increased state of neuroinflammation in the aged brain, contributing significantly to cognitive decline (Sweeney et al. 2018b, 2019). Importantly, our results show that age‐related BBB disruption can be partially reversed by employing senolytic treatment paradigms. These observations imply a causal relationship between CMVEC senescence and BBB disruption. Supporting this, our recent investigations have shown that both gamma irradiation and chemotherapy induce CMVEC senescence in the mouse brain, mirroring aging phenotypes, including BBB disruption (Ahire et al. 2023; Gulej et al. 2023a). Notably, senolytic treatments effectively ameliorate BBB disruption caused by both brain irradiation and chemotherapy (Ahire et al. 2023; Gulej et al. 2023a). Further evidence of the causal link between senescence and BBB integrity comes from studies on budding uninhibited by benzimidazole‐related 1 (BubR1) hypomorphic (BubR1^H/H^) mice, which exhibit accelerated cellular senescence and consequent BBB disruption (Yamazaki et al. 2016). Additionally, experimentally induced senescence in cultured endothelial cells correlates with impaired barrier function (Ya et al. 2023). The underlying mechanisms by which CMVEC senescence contributes to BBB disruption likely involve modifications in tight junctions, dysregulation of specific tight junction proteins, changes in transcellular transport, and/or indirect effects driven by inflammatory mediators released by the senescent cells, part of the SASP (Ya et al. 2023; Banks et al. 2021). Moreover, scRNA‐seq‐derived cell–cell communication analysis reveals a weakened interaction between endothelial cells and pericytes, particularly through disrupted PDGFB/PDGFR‐β signaling, which is critical for capillary stabilization and survival. The breakdown of this essential signaling pathway exacerbates capillary loss and increases BBB permeability, further compromising neurovascular function.

Within the cerebral microcirculation, endothelial cells are interconnected through gap junctions, facilitating the transfer of solutes and cytoplasmic signals between them. This network forms a cohesive functional syncytium, allowing a single senescent endothelial cell to influence the function and phenotype of adjacent CMVECs directly (de Wit et al. 2006; Hautefort et al. 2019). Furthermore, senescence can spread through the microcirculation as adjacent cells are exposed to SASP factors released by senescent endothelial cells, a phenomenon termed paracrine senescence (Acosta et al. 2013). Consequently, an increased number of senescent endothelial cells in the aged brain is likely to adversely affect a substantial portion of the cerebral microcirculatory network In contrast, targeting and eliminating senescent CMVECs using senolytic therapies is anticipated to offer protective benefits across the cerebral microcirculation.

We hypothesize that the improvement of NVC responses, BBB integrity, and increased capillary density plays a key role in the cognitive improvements observed following senolytic treatments in aged mice (Tarantini et al. 2021) and models of accelerated microvascular senescence (Ahire et al. 2023; Yabluchanksiy et al. 2020). Multiple lines of evidence bolster this hypothesis. Clinical studies have demonstrated that BBB disruption (Sweeney et al. 2018a, 2018b, 2019; Montagne et al. 2015; Nation et al. 2019; Kerkhofs et al. 2021) and impaired NVC (Sorond et al. 2013, 2011) are predictive of cognitive decline in humans. Supporting this, preclinical research has established causal relationships between BBB disruption, neurovascular dysfunction, microvascular rarefaction, and cognitive deficits (Toth et al. 2017; Sweeney et al. 2019; Tarantini et al. 2015). The processes through which reduced capillarization and diminished NVC responses detrimentally affect brain health include the onset of ischemic injury in vulnerable areas, especially during periods of heightened metabolic demand. The mechanisms through which leakage of plasma‐derived factors through the compromised BBB impairs neuronal function are likely complex and varied. One significant pathway may involve the activation of microglia, leading to the induction of neuroinflammation, synaptic dysfunction, and white matter injury (Sweeney et al. 2018a, 2018b, 2019).

Besides the strength of the study, some limitations need to be mentioned. The study is restricted to single neuronal activity measurement, and since there was no significant difference between young (4–6 months) and middle‐aged (15–17 months) animals, we focused on the later “aged” time point at a higher time resolution, which showed much more significant changes following the accumulation of senescence. Similarly to the previous, behavioral measurements can be validated with other methods like Y‐maze and T‐maze. The current study is focused on endothelial senescence, and it does not further evaluate the senescence of other cell types.

In conclusion, our research reveals that senolytic treatments yield significant microvascular protective benefits in aging mice, which is likely to contribute to cognitive improvements. These results advocate for the exploration of senolytic strategies as a preventative approach for VCI and dementia in older adults. With numerous clinical trials currently exploring various senolytic regimens for a broad spectrum of diseases already in progress, the prospect of conducting clinical trials aimed at preserving cognitive function in older adults vulnerable to VCI and dementia appears both promising and practical.

## Author Contributions

The foundational concept of this study was developed by A.C. and Z.U., while the overall design and interpretation of data were collaboratively contributed to by all authors. A.N‐T., B.C., R.G., S.C., M.N., and J.D. were responsible for performing surgeries and monitoring the animals post – operatively. R.G., K.V.K., P.B., and S.S. conducted flow cytometry and prepared samples for sequencing. Neurovascular coupling assessments were carried out by S.S.C., S.N., and S.T., B.C., A.N‐T., R.P., and J.D. were tasked with evaluating blood – brain barrier permeability and microvascular density, in addition to analyzing the data. Behavioral evaluations and their subsequent data analysis were expertly handled by S.S., P.M., A.Y., S.N., A.U., and S.S.C. Image analysis was conducted by A.N‐T., R.Y.N., A.U., S.N., S.S., S.S.C., A.Y., and P.M. Single cell data was analyzed by T.O. and T.K. The initial manuscript draft was co – authored by A.N‐T., A.C., and Z.U., with all authors actively participating in the manuscript's revisions. This collective effort culminated in a unanimously approved final manuscript.

## Conflicts of Interest

The authors declare no conflicts of interest.

## Supporting information


Data S1.

## Data Availability

The data that support the findings of this study are available on request from the corresponding author. The data are not publicly available due to privacy or ethical restrictions.
