# Supplementary material for: Senescent Endothelial Cells in Cerebral Microcirculation Are Key Drivers of Age‐Related Blood–Brain Barrier Disruption, Microvascular Rarefaction, and Neurovascular Coupling Impairment in Mice
Source: Aging Cell. 2025 Apr 1;24(7):e70048. doi: 10.1111/acel.70048 (PMC12266767; doi:10.1111/acel.70048)
Supplement: Supplementary file 1 — Data S1. [file ACEL-24-e70048-s001.docx]

**SUPPLEMENTAL FIGURES**

**
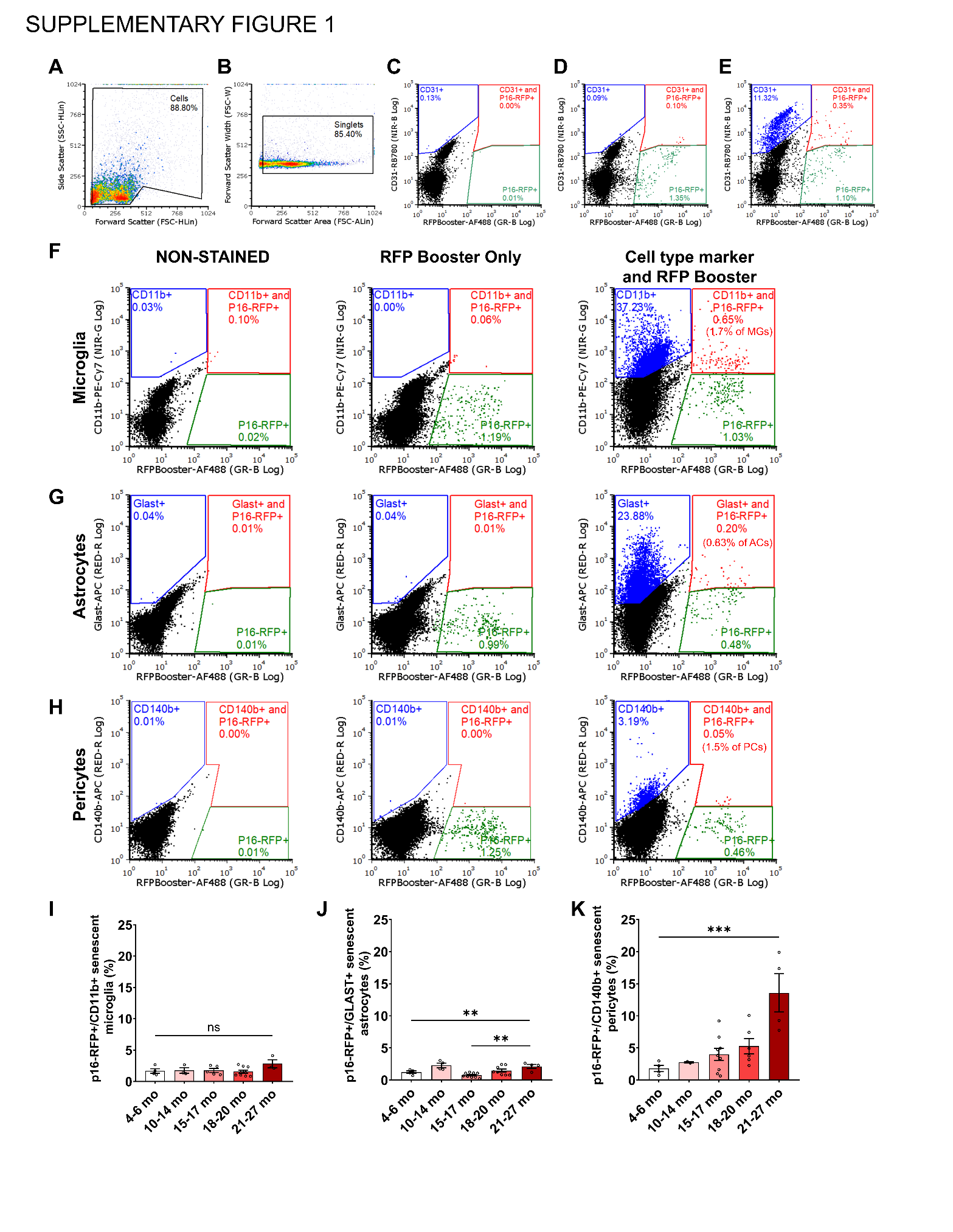
**

**Supplemental Figure 1. Flow cytometry gating strategy for endothelial senescence assessment and evaluation of senescence burden in other brain cell types.** This figure outlines the flow cytometry gating strategy employed to evaluate endothelial cell senescence within the brain microvasculature of p16-3MR senescence reporter animals. Through flow cytometric analysis, we aimed to quantify the burden of senescent cells by simultaneously labeling cells with CD31 and p16-RFP. The detection of the RFP signal was enhanced by employing a custom-synthesized RFP-Booster conjugated to Alexa Fluor 488. (A-D) Panels A and B illustrate the step-by-step gating strategy used to isolate endothelial cells for senescence analysis. Initially, cellular debris was excluded to ensure the purity of the analysis. This was followed by the identification and selection of single cells to avoid doublets or aggregates, thereby focusing on individual cellular events. Panel C presents a non-stained control sample and Panel D shows the RFP-Booster-only control. These controls were used to precisely identify (gate) RFP+/CD31+ and RFP+/CD31- cells, allowing for quantification of senescent endothelial cells (Panel E). This gating procedure allows for precise quantification of senescent cells, providing insights into the cellular aging processes within the brain's microvascular network. Importantly, the same gating strategy was used to quantify senescence burden within microglia, astrocytes, and pericytes. F) Representative plots of non-stained, RFP-Booster stained and CD11b and RFP-Booster-stained cells. I) Quantification of the microglial senescence based on the flow cytometry data. G) Representative plots of non-stained, RFP-Booster stained and GLAST and RFP-Booster-stained cells. J) Quantification of the astrocyte senescence based on the flow cytometry data. H) Representative plots of non-stained, RFP-Booster stained and CD140b and RFP-Booster-stained cells. K) Quantification of pericyte senescence based on the flow cytometry data. All values expressed as mean±SEM (n≥4 for each group). **P<0.01, ***P <0.001 with ANOVA, followed by Tukey post hoc test.


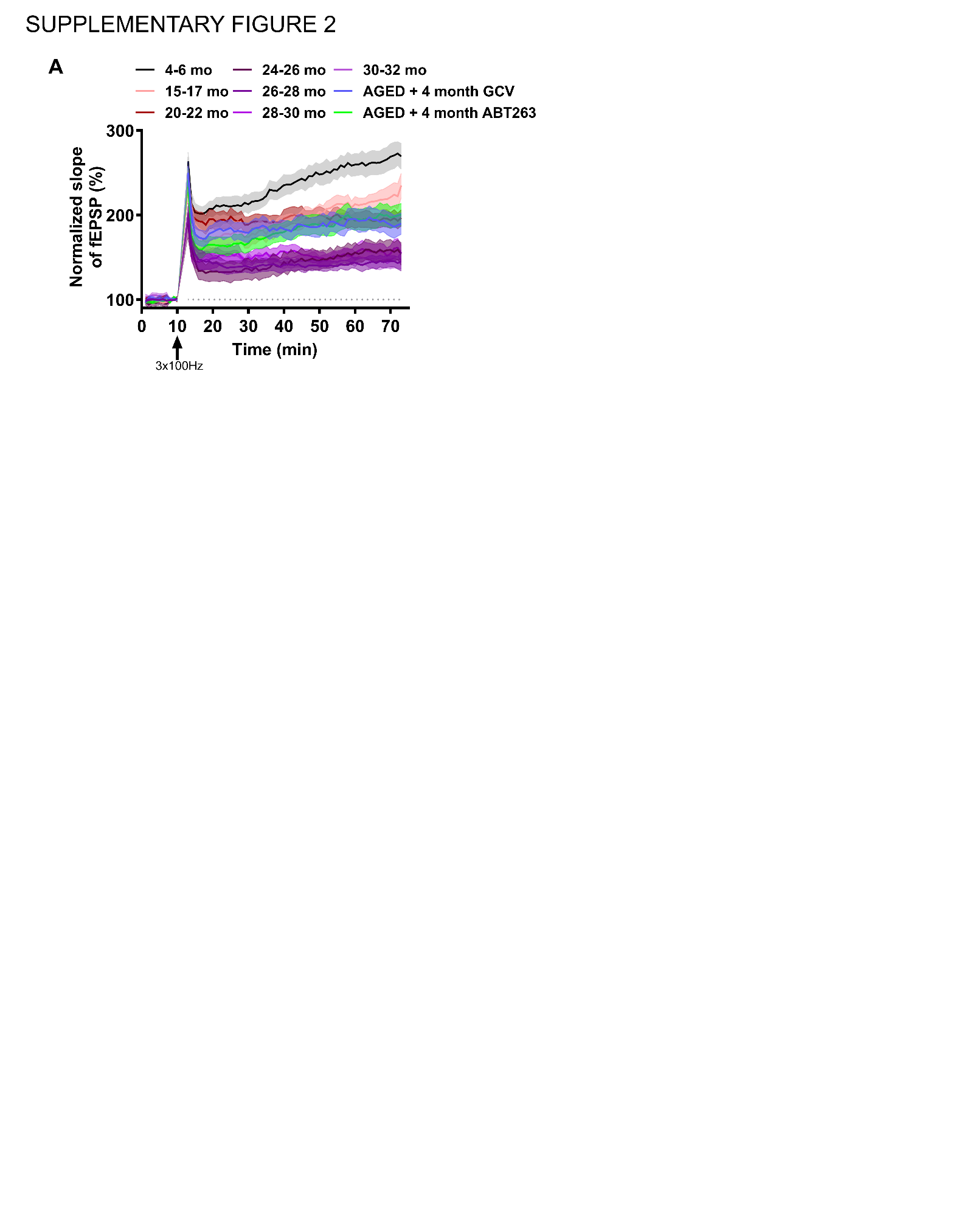


**Supplemental Figure 2.** A) Time course of the normalized fEPSP slopes measured at the hippocampal CA1 synapses from mice of different ages and senolytic treatments. Long term potentiation (LTP) was induced using high-frequency stimulation train of 100 pulses at 100 Hz in all groups of mice (n≥12 slices from 5-6 mice per group). Each data point was obtained as the average of two successive test responses. The vertical arrow indicates the period of three 100 Hz stimulation. Data are shown as mean ± SEM.


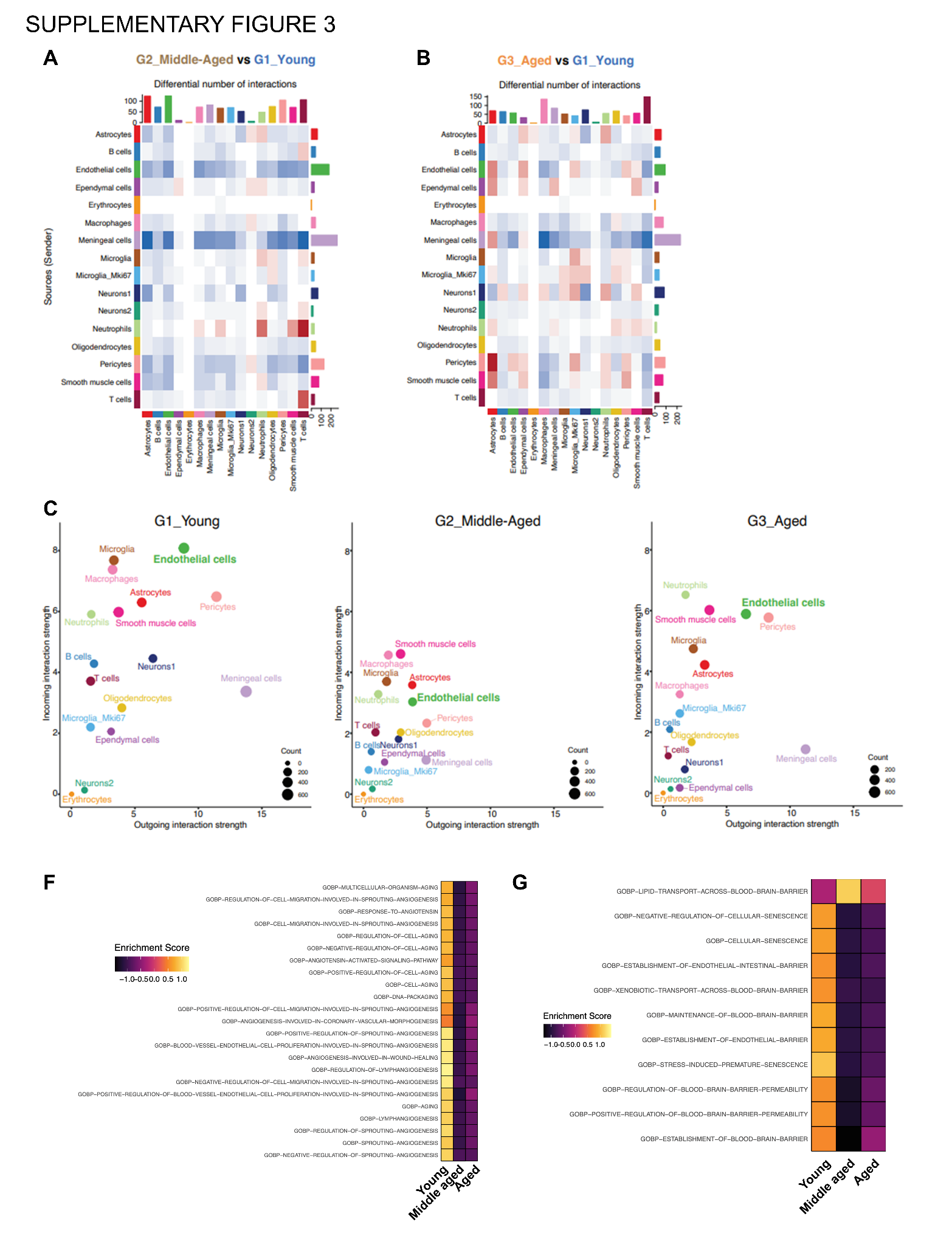


**Supplemental Figure 3. Age-related differences in cell-cell interactions, network connectivity, and functional enrichment across brain cell populations. (**A, B) Heatmaps showing the differential number of cell-cell interactions between groups: (A) Middle-aged (G2) vs. young (G1) samples. (B) Aged (G3) vs. young (G1) samples. The x-axis represents target cell types, and the y-axis represents source cell types. The color scale indicates the strength and number of differential interactions, with red representing increased interactions and blue representing decreased interactions in the older groups compared to young controls. (C) Bubble plots display network centrality analysis for young (G1), middle-aged (G2), and aged (G3) groups. The x-axis shows outgoing interaction strength, and the y-axis shows incoming interaction strength for each cell type. The size of the bubbles reflects the total number of interactions (count), highlighting the central role of endothelial cells, microglia, and smooth muscle cells in aged brains. (F, G) Heatmaps illustrating functional enrichment analysis of age-associated pathways: (F) Enrichment of gene ontology (GO) terms related to cell adhesion, immune response, and vascular regulation across young, middle-aged, and aged groups. (G) Enrichment of pathways linked to blood-brain barrier integrity, cellular senescence, and neurovascular dysfunction. The color scale represents enrichment scores, with orange/purple indicating higher/lower pathway enrichment, respectively. This figure highlights the progressive alterations in cellular communication, network connectivity, and vascular-related functional pathways with aging, particularly involving endothelial and immune cell interactions.
